# On the use of envelope following responses to estimate peripheral level compression in the auditory system

**DOI:** 10.1038/s41598-021-85850-x

**Published:** 2021-03-26

**Authors:** Gerard Encina-Llamas, Torsten Dau, Bastian Epp

**Affiliations:** Hearing Systems Section, Department of Health Technology, Technical University of Denmark (DTU), 2800 Kongens Lyngby, Denmark

**Keywords:** Neuroscience, Physiology, Biomarkers, Health care, Medical research

## Abstract

Individual estimates of cochlear compression may provide complementary information to traditional audiometric hearing thresholds in disentangling different types of peripheral cochlear damage. Here we investigated the use of the slope of envelope following response (EFR) magnitude-level functions obtained from four simultaneously presented amplitude modulated tones with modulation frequencies of 80–100 Hz as a proxy of peripheral level compression. Compression estimates in individual normal hearing (NH) listeners were consistent with previously reported group-averaged compression estimates based on psychoacoustical and distortion-product oto-acoustic emission (DPOAE) measures in human listeners. They were also similar to basilar membrane (BM) compression values measured invasively in non-human mammals. EFR-based compression estimates in hearing-impaired listeners were less compressive than those for the NH listeners, consistent with a reduction of BM compression. Cochlear compression was also estimated using DPOAEs in the same NH listeners. DPOAE estimates were larger (less compressive) than EFRs estimates, showing no correlation. Despite the numerical concordance between EFR-based compression estimates and group-averaged estimates from other methods, simulations using an auditory nerve (AN) model revealed that compression estimates based on EFRs might be highly influenced by contributions from off-characteristic frequency (CF) neural populations. This compromises the possibility to estimate on-CF (i.e., frequency-specific or “local”) peripheral level compression with EFRs.

## Introduction

Hearing impairment is one of the most common chronic troublesome conditions for elderly people in the ageing Western societies^1^, and can imply a significant functional limitation^2^. Although cell regeneration techniques are under development^3,4^, their success is dependent on reliable and precise diagnostic measures that can differentiate between types of peripheral cellular damage in individual patients. A reliable estimate of cochlear compression could be used to assess differential damage of inner hair cells (IHC) and outer hair cells (OHC)^5^. An important characteristic of the healthy mammalian auditory system is the compressive transformation of the large dynamic range of input sound pressure levels (SPL) to a narrower range of levels that can be processed by the sensory cells. Part of this compressive transformation is a consequence of the processing by the OHCs in the cochlea. Although there is still some controversy about the precise mechanism underlying OHC function (e.g.,^6–10^), it is broadly accepted that OHC electro-motility provides a level-dependent gain to the movement of the BM in the healthy cochlea. This leads to a high sensitivity to low-level sounds and a compressive input/output (I/O) function at the characteristic place of the BM for tonal stimuli^11^. In addition to increasing sensitivity, OHC function has also been associated with high frequency selectivity and a normal loudness growth with level^12^.

Invasive physiological recordings in living non-human mammals allow precise measures of place-specific BM velocity-level functions using pure-tone stimuli (e.g.,^13–17^). For a pure tone, the envelope of the resulting travelling wave shows a maximum at one specific cochlear place. Magnitude-level functions measured at or near this place (“on-CF”) show a compressive growth of BM velocity with increasing SPL, consistent with the idea of a level-dependent amplification. Basal and apical to this place (“off-CF”), the magnitude-level functions show a linear growth (Figs. 6 and 7 in^15^). The combination of non-linear on-CF and linear off-CF magnitude-level functions leads to a level-dependent BM excitation pattern with sharp tuning at low levels and broader tuning at higher levels. In the case of OHC dysfunction, on-CF magnitude-level functions show reduced compression. This leads to a lower on-CF response amplitude and a less level-dependent tuning of the BM excitation pattern^18^. Direct measurements of BM velocity are not possible in-vivo in humans due to ethical considerations. Instead, behavioural measurements using forward-masking paradigms (e.g.,^19–24^) as well as objective measurements based on distortion-product oto-acoustic emission (DPOAE) amplitudes (e.g.,^25,26^) have been considered to estimate BM compression in humans.

Oto-acoustic emissions (OAE) are low-level sounds recorded in the ear canal^27^ generated in the cochlea as a side-effect of the non-linear processing induced by the OHC’s electro-motility. The slope of DPOAE magnitude-level functions has been proposed as an estimate of cochlear compression^25^. Group-averaged DPOAE slopes in NH listeners showed a compressive growth with increasing stimulus level. In hearing-impaired (HI) listeners, group-averaged DPOAE slopes showed a reduced range and amount of compression, together with a higher stimulus level required to evoke a measurable DPOAE^26^. However, these studies reported high variability of DPOAE slopes across individual listeners^28^, compromising their predictive value for an individual listener^25^.

The envelope following response (EFR) represent another objective and non-invasive measure to investigate auditory function. EFRs, also referred to as auditory steady-state response (ASSR), are gross electroencephalographic (EEG) potentials elicited by populations of neurons that respond synchronously (phase-locked) to the envelope of an acoustic stimulus. The EFR amplitude varies depending on the modulation frequency, with a predominant peak at ${40}\,\hbox {Hz}$ (the so-called ${40}\,\hbox {Hz}$ potential) and a smaller peak around 80–100 Hz (the ${80}\,\hbox {Hz}$ potential)^29^. It has been suggested that, when the traditional clinical vertical electrode montage is used, EFRs to 80–100 Hz modulations are mainly generated by brainstem-midbrain sources^30^, although cortical sources may also contribute^31–33^. EFRs to ${40}\,\hbox {Hz}$ modulations are thought to have more dominant sources from cortical stages^30^. Due to their narrow bandwidth, multiple sinusoidally amplitude modulated (SAM) tones have been used to record EFRs evoked at multiple cochlear regions simultaneously to speed up clinical assessment^34–36^. While the carrier frequency of each SAM tone determines the cochlear region to be excited, different modulation frequencies produce different peaks in the recorded EEG spectrum^37^, making it possible to separate the responses in the frequency domain. It has been demonstrated that EFRs can be recorded using four simultaneous SAM tones modulated between 80–100 Hz^35^, a technique currently implemented in clinical systems to estimate hearing thresholds (e.g.,^38^). At supra-threshold levels, EFR magnitudes as a function of stimulus level (EFR magnitude-level functions) elicited from single SAM tones show a monotonic growth^39–41^. It was shown that EFR magnitudes evoked by multiple SAM tones presented simultaneously, separated one octave apart and modulated around 80–100 Hz, grow monotonically up to about 60 dB SPL followed by a plateau^42^. EFR magnitudes are also influenced by the modulation depth. For a fixed stimulus level and modulation frequency, the magnitude of the EFR drops when reducing the modulation depth of the stimulus^39,41,43^. Given that the modulation depth of a SAM tone is reduced when passed through a compressive non-linearity (e.g., on-CF BM processing), the EFR magnitudes may be lower for a system with high compression compared to a system with low compression (see^44^). Consequently, the slope of the EFR magnitude-level function as a function of stimulus level should be shallower for a more compressive system compared to a less compressive system, potentially revealing the compressive growth of the BM.

In the present study, EFR magnitude-level functions elicited by four simultaneous SAM tones modulated at 80–100 Hz were measured in NH and HI listeners. DPOAE magnitude-level functions were also measured in the same NH listeners for comparison. Even though EFRs evoked by a 80–100 Hz modulation may be predominantly elicited at brainstem-midbrain auditory stages^30,45,46^, the compression estimates derived from the slope of the EFR magnitude-level functions may reflect a combination of various compressive processes at cochlear and retro-cochlear levels (i.e., brainstem-midbrain and even primary cortex^31–33^). Modulation frequencies of 80-100 Hz were chosen to find a balance between strong EFR amplitudes and as peripheral as possible EFR generating sources. Potential variations of generating sources with increasing stimulus levels were investigated by analysing the phases (or latencies) of the recorded EFRs. Assuming that all sources of compression beyond cochlear processing are not, or only minimally affected by OHC dysfunction, a change in BM compression will be reflected at more central levels in the auditory pathway (i.e., brainstem-midbrain). Such a change will then be represented in the magnitude of the EFR. This assumption could be compromised by the presence of cochlear synaptopathy (CS), the loss of the synaptic terminals of peripheral auditory nerve (AN) neurons innervating the IHCs^47^. CS could lead to a change in the slope of EFR magnitude-level functions^41,48^. This assumption was investigated using computational simulations for realistic CS profiles^49^. The aim of the present study was to investigate whether peripheral level compression can be estimated simultaneously at four different carrier frequencies using EFR magnitude-level functions. It was anticipated that the amount of compression estimated through the EFR will be higher in NH listeners compared to HI listeners due to the reduced contribution of BM compression in the HI listeners with sensorineural hearing loss. Prior to proposing the potential clinical use of the slope of the EFR magnitude-level functions as an estimate of peripheral compression, the stability of the measure must be demonstrated. An EFR test–retest repeatability analysis was performed in NH listeners both for individual EFR data points and for EFR slopes. In addition to the analysis of the experimental data, a well-established phenomenological computer model of the AN^50^ was used to investigate the mechanisms underlying the peripheral contributions to the EFRs generation. Due to the frequency-specific (on-CF) nature of BM compression, special attention was dedicated to analyse the effect of off-CF AN contributions to the compression estimates derived from the simulated EFR magnitude-level functions. Effects of OHC and IHC dysfunction on the EFR-based compression estimates, as well as AN fibre loss, were systematically investigated within the modelling framework. Compression estimates derived from EFRs were compared to compression estimates from DPOAEs in the same NH listeners.

## Methods

### Listeners

Twenty adult listeners (10 females, 34.0 ± 15.9 years) participated in this study. Listeners were separated into groups of 13 NH (8 females, 24 ± 3.2 years) and 7 HI (2 females, 56.2 ± 12.7 years). All NH listeners had audiometric thresholds below ${15}\,\hbox {dB}$ hearing level (HL) at octave frequencies between 125 and ${8000}\,\hbox {Hz}$. All HI listeners were selected to have normal-hearing thresholds ($\le$ ${20}\,\hbox {dB}$ HL) below ${4000}\,\hbox {Hz}$ and a mild hearing impairment at ${4000}\,\hbox {Hz}$ and above, with audiometric thresholds between 20 and ${45}\,\hbox {dB}$ HL.

All participants provided informed consent and all experiments were approved by the Science-Ethics Committee for the Capital Region of Denmark (reference H-3-2013-004). The experiments were carried out in accordance with the corresponding guidelines and regulations on the use of human subjects. The listeners were economically compensated for participating in the experiments.

### Apparatus

The EFR and DPOAE recordings were performed in a dark, soundproof and electrically shielded booth, where the participants were seated in a comfortable reclined armchair. The participants were instructed to close their eyes and relax to avoid moving and were allowed to sleep. The recording and data analysis routines were implemented in MATLAB (The MathWorks, Inc., Natick, Massachusetts, USA). All acoustic stimuli were generated in MATLAB and presented using PLAYREC 2.1 (Humphrey, R., www.playrec.co.uk, 2008–2014) via a RME Fireface UCX soundcard (sampling rate $f_{\mathrm {s|sound}}=48$ kHz, 24 bits). The analogue acoustic signal was passed to a headphone buffer (HB7, Tucker–Davis Technologies) with a gain of ${-6}\,\hbox {dB}$ (stimulus levels > ${55}\,\hbox {dB}$ SPL) or ${-27}\,\hbox {dB}$ (stimulus levels $\le$ ${55}\,\hbox {dB}$ SPL). The attenuated signal was presented through a pair of ER-2 insert earphones (Etymotic Research Inc.) mounted on an ER-10B+ low-noise DPOAE microphone probe (Etymotic Research Inc.) with ER10-14 foam eartips, from which the DPOAE was recorded.

EFRs were recorded using a Biosemi ActiveTwo system (sampling rate $f_{\mathrm {s|{EFR}}}=8192$ Hz, speed mode 6, 24 bits). Five active pin-type electrodes were used. Three electrodes were mounted at positions P10, P9 (right and left extremes of the parietal coronal line) and Cz (vertex) following the 10-20 system^51^. The remaining two electrodes (common mode sense (CMS) and driven right leg (DRL)) were placed at the centre of the parieto-occipital coronal line (on either side of electrode POz). The electrodes CMS and DRL form a feedback loop that replace the “ground” electrode (the zero) in conventional EEG systems^52^. Conductive electrode gel was applied and the offset voltage was stabilised at $< {20} \,{\hbox {m} \hbox {V}}$ for each electrode. The recorded EEG signals were hardware low-pass filtered by the EEG amplifier with a bandwidth limit of $\frac{1}{5^{\mathrm{th}}}$ of the sampling frequency (anti-aliasing filter) and down sampled by a factor of 2 by the Biosemi software. The EEG data were stored to hard disk. The results shown in this study represent the Cz-P10 potential in response to right-ear stimulation, and the Cz-P9 potential in response to left-ear stimulation.

DPOAE were recorded using the same stimulus presentation apparatus as for the EFR measurements, but the ER-10B+ ear probe was connected to the ER-10B+ pre-amplifier (with a gain of ${20}\,\hbox {dB}$). The microphone signal was bandpass filtered using a cascade of a high-pass filter (Rockland model 852, Butterworth ${48}\,\hbox {dB}$/octave, cut-off frequency 100 Hz) and a low-pass filter (cut-off frequency ${9}\,\hbox {kHz}$, otherwise identical to the high-pass filter). The recorded signal was digitised using the sound card at $f_{\mathrm {s|sound}}=48$ kHz and 24 bits resolution and stored to hard disk.

### EFR recordings

The EFR data were recorded in two sessions. In the first session (approx. two hours in duration), the EFR magnitude-level functions were recorded in the NH listeners using input levels in the range from 20 to ${80}\,\hbox {dB}$ SPL, in steps of ${5}\,\hbox {dB}$. The second recording session (approx. 45 minutes in duration) took place on a different day usually about one month later than the first session. Three input levels (35, 55 and ${70}\,\hbox {dB}$ SPL) were recorded again in the same NH listeners to evaluate the test–retest repeatability of the results. In all NH listeners, the right ear was stimulated. In the HI group, the multi-frequency recording was carried out in the level range from 30 to ${80}\,\hbox {dB}$ SPL, in steps of ${5}\,\hbox {dB}$. Here, the recording ear was chosen depending on the individual listener’s audiogram, such that the amount of sensitivity loss due to the hearing impairment was as similar as possible within the group. There was no second recording session to evaluate repeatability for this subject group.

A multi-frequency stimulus consisting of four SAM tones was used. The SAM tones had carrier frequencies of 498, 1000, 2005 and ${4011}\,\hbox {Hz}$ (referred to as 500, 1000, 2000 and ${4000}\,\hbox {Hz}$ throughout this work) modulated at 81, 87, 93 and ${98}\,\hbox {Hz}$, respectively. The modulation depth was set to $\mathrm {m}=85 \%$. The four SAM tones were calibrated individually to the desired root mean squared (RMS) value using a B&K 4157 ear simulator, and were added later together (resulting in a final stimulus level that was ${6}\,\hbox {dB}$ higher than that of each individual SAM tone). The stimuli were digitally generated as 1-s long epochs and continuously presented to the listener, where a trigger signal marked the beginning of a new epoch for later averaging. The total stimulus duration depended on the stimulus intensity to achieve a statistically significant EFR signal-to-noise ratio (SNR), based on a pilot study. Table 1 shows the stimulus duration used for each input level in the EFR recordings.

**Table 1:** Duration of EFR stimuli for each input level used in the NH listeners.

Input level (dB SPL)	20	25	30	35	40	45	50	55	60	65	70	75	80
Duration (min)	12	12	11.2	10.13	8.53	8.53	7.73	7.2	7.2	6.67	5.6	5.6	5.6

The recorded EEG data were filtered using a fourth-order Butterworth digital band-pass filter with corner frequencies of 60 and ${400}\,\hbox {Hz}$, applied serially in forward and backward direction to yield zero phase. All recorded epochs with a maximum absolute amplitude that exceeded a voltage threshold of ${80}\,{\upmu \,\hbox {V}}$ in any of the channels were rejected to remove artefacts and noisy events from the average pool. Sixteen 1-s long epochs of EEG data were concatenated forming a trial to achieve a higher frequency resolution in the EFR spectrum analysis. In order to increase the SNR, the 16-s long trials were ensemble weighted averaged, where the inverse of the variance on each 1-s long epochs was used as weights^53^. A fast Fourier transform (FFT) was performed on the averaged waveform and the EFR magnitude and phase values were read out from each of the modulation frequency bins of interest. Unwrapped EFR phases (in degrees) were delayed by ${90}\,^{\circ }$ to compensate for sine starting phases in the stimuli, and converted to latency dividing by $360 \cdot f_m$, where $f_m$ is the modulation frequency.

### DPOAE recordings

DPOAE were measured (except for listener NH03, who could not participate) during the second recording session. DPOAE were measured using a sweeping technique^54^ and the non-linear distortion source was unmixed using a time-windowing method^55,56^. The sweeping primaries consisted of two upward sweeps of equal level with a duration of 10-s and a sweep rate of half an octave per second^54^. Primary $f_{2}$ ranged from 250 to ${8000}\,\hbox {Hz}$, while keeping the frequency ratio constant at $\frac{f_{2}}{f_{1}}=1.22$. The distortion-source DPOAE magnitude-level functions of the $2f_{1}-f_{2}$ component were recorded at primary levels of 30 to ${65}\,\hbox {dB}$ SPL, in steps of ${5}\,\hbox {dB}$. The acoustic waveforms recorded in the ear canal were analysed in overlapping, windowed time frames (Hanning window of 24000 samples with a step size of 600 samples) using a least-squares-fit procedure which estimated the magnitude and phase of a sinusoid to the expected DPOAE frequency component^54^.

The recorded sweeps were averaged to increase the SNR of the DPOAE. Prior to averaging, noisy frames were rejected in an online procedure that estimated the SNR in a pair of time frames. The background noise was estimated by averaging two consecutive frames after inverting the phase of the second one by ${\pi }\,{\hbox {rad}}$ to remove deterministic components. The SNR estimation was defined as the difference between the magnitudes of the DPOAE non-linear distortion component and the estimated background noise. Two stopping rules were defined. The recording ended when the SNR in all four frequency bins of interest (0.5, 1, 2 and ${4}\,\hbox {kHz}$) were larger than ${10}\,\hbox {dB}$ or when 8 pairs of sweeps had been recorded. For an individual listener, the recording of the complete DPOAE magnitude-level functions lasted approximately ${20}\,{\hbox {min}}$.

### Fitting functions

To estimate compression from the slope of each individual EFR magnitude-level function, a piecewise linear function with two segments was fitted using a non-linear least squares method described by:

1 $$\begin{aligned} f(L_{s}) = {\left\{ \begin{array}{ll} s_{1} \cdot \left( L_{s} - b_{x} \right) + b_{y} &{} \quad \text {if}\,\, L_{s} < b_{x} \\ s_{2} \cdot \left( L_{s} - b_{x} \right) + b_{y} &{} \quad \text {if}\,\, L_{s} > b_{x} \end{array}\right. } \end{aligned}$$

where $L_{s}$ represents the stimulus input level, $s_{1}$ the lower slope, $s_{2}$ the upper slope and $b_{x}$ and $b_{y}$ represent the value on the abscissa and the ordinate at the break-point, respectively. Motivated by the BM I/O function characteristics observed either in direct animal physiological recordings (e.g.,^15^) or used for human psychoacoustical estimates of cochlear compression (e.g.,^12,19–21^), the lower slope was forced to be larger than the upper slope (i.e. $s_{1} > s_{2}$). Otherwise a first-order polynomial (single slope) was used: $f(L_{s}) = s \cdot L_{s} + a$, where *s* is the slope and *a* the intercept. In case that a first-order polynomial model was found to provide a better fit than the two-slopes piecewise functions (based on an adjusted $R^2$ statistic), this simpler model was used. The two-slopes piecewise function was only considered if at least 3 significant data points were present on each segment of the fitting function. Only EFR readings significantly above the background noise floor were included in the fitting procedure.

For individual DPOAE magnitude-level functions, a simple first-order polynomial model was fitted to the statistically significant data points. The value of the slope of the fitted model (*s*) was considered to be the cochlear compression estimate, as proposed in^25^.

### Statistical analysis

Statistically significant EFRs were identified by means of a F-test that compared the spectral power at the modulation frequency (EFR frequency) to the noise power in the range of ${3}\,\hbox {Hz}$ below and above the modulation frequency^29,57^. The power ratio (*F-ratio*) was calculated as the power in the EFR frequency bin divided by the averaged power in ${3}\,\hbox {Hz}$ below and above the modulation frequency (96 bins). The probability (*p*) of the EFR power being different from the noise power can be calculated as $1-F$, with F representing the cumulative distribution function of the power ratio. The F-test was defined to be positive if $p \le 0.01$ (F critical value $\le$ 4.8333, $\mathrm{SNR}> {5.84}\,\hbox {dB}$), implying that the EFR frequency was statistically significant from the noise estimate. The F-test was custom implemented in MATLAB. In the case of DPOAE recordings, statistical significance was determined when the SNR of the DPOAE for a given frequency was $\ge {10}\,{\hbox {dB}}$.

In order to test whether the estimated EFR slopes, DPOAE slopes and EFR breakpoints from two different conditions (frequency or hearing status) were statistically different from each other, a two-sample permutation test for equality of the means was used^58,59^. The test evaluates the hypothesis that the estimated parameter for two conditions were a random partition all data added together, against the alternative hypothesis that the estimated parameter from one condition were part of a population with a different mean than the other condition. The test was performed using 50,000 permutations using the Permute package implemented in Python^60^. P-values were corrected for multiple comparisons using the false discovery rate method^61^.

The dependence of EFR latency on stimulus level was analysed using a linear mixed-effects model (LMM) (“lme4” R-package, v1.1.23^62^ fitted using the “lmerTest” R-package v3.1.2^63,64^) implemented in R 3.2.2 (R Core Team 2015). Stimulation level and frequency were treated as fixed effects, while listeners, hearing status (i.e., NH or HI) and the interactions between level and frequency and between hearing and frequency were treated as random effects. F-tests using the Satterthwaite’s method to approximate the denominator degrees of freedom were used to calculate the p-values for the fixed effects. The p-values for the random effects were calculated based on a likelihood ratio test^64^.

### EFR test–retest repeatability

The accuracy of the fitted slope to the EFR magnitude-level function will depend on the test–retest variability of each individual EFR data point. In order to have an estimate of the measurement variability, the repeatability of individual EFR magnitudes at three stimulus levels was assessed using a Bland–Altman analysis^65^. Data was also analysed using a one-way, random-effects, single-rater intra-class correlation (ICC) coefficient^66,67^, interpreted using the guidelines provided by^68^. Normality was ensured by means of a visual inspection of the corresponding quantile–quantile (Q-Q) plots^69^ and by computing a Shapiro–Wilk normality test^70^ (not shown). In the Bland–Altman analysis, the test–retest difference values (i.e., the value of the retest subtracted to the value of the test) were plotted against the mean response amplitude between two test runs. This method defines the limits of agreement (LoA) as 1.96 times the standard deviation of the differences. The method proposed by^71^ was used to compute ${95}\,\%$ confidence interval (CI) for the mean of the differences and for the upper and lower LoA. The same repeatability analysis was performed on the EFR slopes. At frequencies 500, 1000 and ${2000}\,\hbox {Hz}$, only the stimulus levels of 35 and ${55}\,\hbox {dB}$ SPL were used both for the test and retest, because the level of ${75}\,\hbox {dB}$ does not belong to the compressive part in the EFR magnitude-level functions. For these frequencies, the EFR slope was estimated as the difference between the EFR magnitudes at 55 and ${35}\,\hbox {dB}$ divided by 20. At ${4}\,\hbox {kHz}$, all three repeated levels were used (35, 55 and ${75}\,\hbox {dB}$), and the EFR slope was estimated by fitting a first order polynomial to the three data points in both the test and the retest. In any case, only statistically significant EFR data points (positive F-test) were considered in the repeatability analysis, discarding those test–retest pairs that contained missing data points.

### AN model

A humanised phenomenological AN model^50^ was used to simulate the activity of the AN. In short, the input acoustic stimulus waveform is processed by a linear filter mimicking the middle-ear filtering. The BM is modelled as a time-varying level-dependent filter-bank where a gain parameter models the effect induced by OHC motion. The IHC transmembrane potential is modelled by a rectifying non-linearity coupled to a low-pass filter that limits phase-locking. Fast and slow power-law functions are used to model offset adaptation and long-term adaptation observed in single AN unit recordings^72^. Short-term onset adaptation is modelled as an adaptive redocking mechanism with four synaptic release sites. The implementation of the AN model is similar to one used in^41^. Each simulation computes a total of 32000 AN fibres, distributed non-uniformly (with more density of fibres at mid CFs based on^73^) through 300 CFs (cochlear segments or IHCs) ranging from ${125}\,\hbox {Hz}$ to ${20}\,\hbox {kHz}$. For each CF, a ${61}\,\%$ of high-spontaneous rate (SR) (HSR) fibres, ${23}\,\%$ of medium-SR (MSR) fibres and ${16}\,\%$ of low-SR (LSR) fibres were considered^74^. Hair-cell dysfunction was implemented by fitting the listener’s audiogram using the *fitaudiogram2* MATLAB function implemented by^72^. This function allows to define the proportion of threshold elevation that is attributed to either OHC or IHC dysfunction.

To simulate EFR magnitude-level functions at the level of the AN, throughout the manuscript referred to as $\mathrm {{EFR}_{{AN}}}$, the same stimulus as the one used in the recordings consisting of four simultaneous SAM tones was presented to the model but of a duration of 1.2-s. The stimuli were calibrated and presented to the AN model ranging from 5 to ${100}\,\hbox {dB}$ SPL in steps of ${5}\,\hbox {dB}$. The spike trains obtained from the independently computed AN neurons for a given CF and fibre type were added together to obtain the summed AN activity at that CF, which is comparable to the peri-stimulus time histogram (PSTH) used to describe data from single neurons in experimental recordings. In order to analyse the steady-state encoding of a modulation, a 1-s long steady-state response, excluding onsets and offsets, was considered. A FFT was performed on the resulting summed AN activity and the magnitude value at the modulation frequency bin was considered the simulated $\mathrm {{EFR}_{{AN}}}$.

In order to visualise which CFs may contribute to the total $\mathrm {{EFR}_{{AN}}}$ response, heatmap plots showing $\mathrm {{EFR}_{{AN}}}$ magnitude as a function of CF and stimulus levels were used (see Fig. 5a–g). In those plots, for each combination of CF and stimulus level, the colour represents the magnitude of the $\mathrm {{EFR}_{{AN}}}$ obtained from the frequency bin corresponding to the modulation frequency of interest (different for each carrier frequency) in the spectrum of the summed AN activity at that CF (the PSTH for that particular CF). The simulated $\mathrm {{EFR}_{{AN}}}$ magnitude-level functions (see Fig. 5h–k) are obtained by summing all the AN simulated activity across CFs and reading the magnitude at the modulation frequency bin from the spectrum of the summed PSTHs. For the analysis done at the on- and off-CF bands, the same procedure is performed over the summed AN activity of all CFs within the definition of on-CF band, or over the summed AN activity of all CFs except the ones of the on-CF band (off-CFs). The on-CF band was defined as the CFs ranging from $\frac{1}{2}$-octave lower and $\frac{1}{3}$-octave higher than the carrier frequency of the SAM tone (a fractional bandwidth of $\approx 28\%$), based on velocity-intensity functions recorded directly in the BM of non-human animal^15^.

## Results

The data reported in this article are publicly available in a Zenodo repository (https://doi.org/10.5281/zenodo.844833)^75^.

### EFR and DPOAE magnitude-level functions

#### Normal-hearing listeners

**Figure 1:**
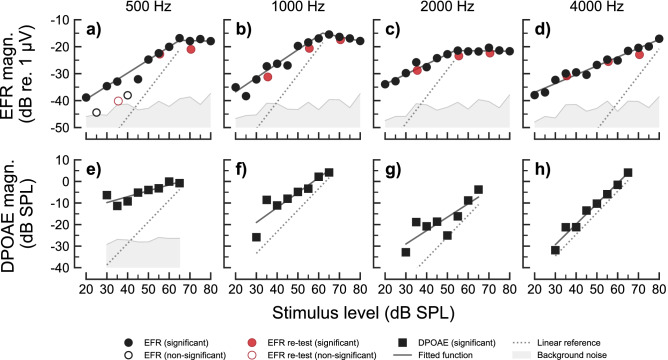
EFR (panels **a**–**d**) and DPOAE (panels **e**–**h**) magnitude-level functions recorded in one representative NH listener (NH01) for the frequencies of ${500}\,\hbox {Hz}$ (panels **a**,**e**), ${1000}\,\hbox {Hz}$ (panels **b**,**f**), ${2000}\,\hbox {Hz}$ (panels c and g) and ${4000}\,\hbox {Hz}$ (panels **d**,**h**). EFR and DPOAE magnitudes are represented as filled circles and squares, respectively, in case of statistically significant responses. Open symbols show statistically non-significant data points. Grey shaded areas show the estimated background noise. In panels (**a**–**d**), black circles indicate EFR magnitudes recorded in the first recording session, and red circles indicate retest EFR magnitudes recorded in the second recording session. Fitted models to significant data points are represented by the solid dark-grey functions. A linear reference with a slope of ${1}\,\hbox {dB/dB}$ is indicated by the dotted line.

**Figure 2:**
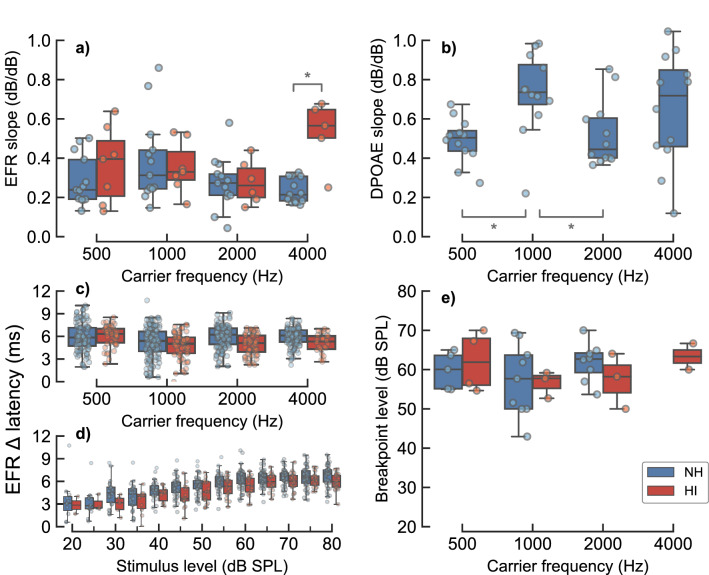
Group statistics of EFR and DPOAE data for NH and HI listeners. Panel (**a**) shows box-plots with the fitted EFR slopes obtained for each carrier frequencies in the NH (blue) and HI (red) listeners. Panel (**b**) shows box-plots with the fitted EFR slopes for same the NH listeners. Panels (**c**,**d**) show EFR latency increment (referenced to the shortest recorded latency) as a function of frequency and stimulus level, respectively. Panel (**e**) shows EFR break-point levels for the different carrier frequencies when the two-slopes piecewise fit was used. The bottom and the top of each box represent the first and third quartiles, respectively, and the horizontal line inside each box represents the second quartile (the median). Whiskers indicate 1.5 times the interquartile range (IQR) of the lower and upper quartiles. The circles depict the raw observations. Statistical significance, based on a two-sample permutation test for equality of the means, is represented by the asterisks, where * corresponds to $p \le 0.05$.

Figure 1 shows the recorded EFR (panels a–d) and DPOAE (panels e–h) magnitude-level functions for one representative NH listener (NH01) for ${500}\,\hbox {Hz}$, ${1000}\,\hbox {Hz}$, ${2000}\,\hbox {Hz}$ and ${4000}\,\hbox {Hz}$, respectively. The complete sets of EFR and DPOAE data for all NH listeners are shown in Supplementary Fig. S1 and in Supplementary Fig. S3, respectively. All EFR magnitude-level functions were found to grow monotonically and compressively (with slopes ${1}\,\hbox {dB/dB}$) for stimulus levels between 20 and 50-${65}\,\hbox {dB}$ SPL. The EFR magnitude-level functions obtained for the carrier frequencies 500, 1000 and ${2000}\,\hbox {Hz}$ (panels a–c in Fig. 1 and Supplementary Fig. S1) showed a different trend than that for the ${4}\,\hbox {kHz}$ carrier (panel d). At 500, 1000 and ${2000}\,\hbox {Hz}$, the EFR magnitudes saturated, or slightly decreased, for stimulus levels above 50-65 dB SPL, leading to a break-point in the magnitude-level function. Figure 2e (blue symbols) shows box-plots indicating the fitted break-point levels at the four carrier frequencies. The median values for the break-point levels varied between 50 to 65 dB SPL, and non of the conditions were statistically different. In contrast, no break-point was observed at ${4}\,\hbox {kHz}$ (hence the absence of a NH box-plot at 4 kHz). At this frequency, the magnitude-level function was found to grow monotonically with a single slope (see also Supplementary Table S1). Figure 2a (blue symbols) shows the estimated EFR slopes. Median values amounted to ${0.24}\,\hbox {dB/dB}$ at ${500}\,\hbox {Hz}$, ${0.31}\,\hbox {dB/dB}$ at ${1000}\,\hbox {Hz}$, ${0.27}\,\hbox {dB/dB}$ at ${2000}\,\hbox {Hz}$ and ${0.21}\,\hbox {dB/dB}$ at ${4000}\,\hbox {Hz}$. The estimated EFR slopes in the NH listeners were not statistically different across frequency.

The distortion-component of the DPOAE magnitude-level functions (panels e–h in Fig. 1 and Supplementary Fig. S3) grew monotonically with increasing stimulation level, generally well represented by the fitted line (see also Supplementary Table S3). The estimated DPOAE slopes showed a large variability across frequency and listeners (Fig. 2b), even though DPOAE sources were unmixed. The median values for the DPOAE slopes in the NH listeners were ${0.5}\,\hbox {dB/dB}$ at ${500}\,\hbox {Hz}$, ${0.74}\,\hbox {dB/dB}$ at ${1000}\,\hbox {Hz}$, ${0.44}\,\hbox {dB/dB}$ at ${2000}\,\hbox {Hz}$ and ${0.72}\,\hbox {dB/dB}$ at ${4000}\,\hbox {Hz}$. The pair-wise comparisons between 500 and ${1000}\,\hbox {Hz}$ (Test statistic = -0.2401 (${95}\,\%$ CI: -0.3788, -0.0971), $p = 0.0146$) and between 1000 and ${2000}\,\hbox {Hz}$ (Test statistic = 0.2082 (${95}\,\%$ CI: 0.0476, 0.3684), $p = 0.0434$) were found to be statistically different.

#### Hearing-impaired listeners

**Figure 3:**
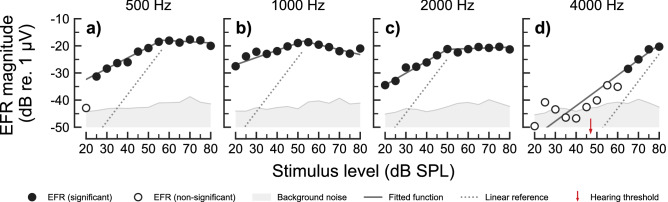
EFR magnitude-level functions recorded in one representative HI listener (HI01) for carrier frequencies of (**a**) ${500}\,\hbox {Hz}$, (**b**) ${1000}\,\hbox {Hz}$, (**c**) ${2000}\,\hbox {Hz}$ and (**d**) ${4000}\,\hbox {Hz}$. Same representation as in Fig. 1 but not including the repeatability measurements. The small red arrow in panel (**d**) indicates the behavioural hearing threshold of the listener at ${4000}\,\hbox {Hz}$ in dB SPL.

Figure 3 shows the EFR magnitude-level functions for one representative HI listener (HI01). The complete set of EFR data for all HI listeners is shown in Supplementary Fig. S2. The EFR magnitude-level functions for 500, 1000 and ${2000}\,\hbox {Hz}$ carrier frequencies (panels a–d) showed similar trends as the ones observed for the NH listeners (shown in Fig. 1 and Supplementary Fig. S1). At ${4}\,\hbox {kHz}$ (Fig. 3d), the EFR magnitudes for stimulus levels up to ${60}\,\hbox {dB}$ SPL were not statistically different from the EEG background noise, whereas significant EFR magnitudes were obtained above ${60}\,\hbox {dB}$ SPL for this particular listener, showing a compressive growth with level (slope < ${1}\,\hbox {dB/dB}$). This frequency is within the region of reduced sensitivity in this listeners’ audiogram (red arrow in panel d). Overall, the EFR magnitudes recorded in some of the HI listeners showed lower SNRs than in the NH listeners, resulting in a larger number of statistically non-significant data points (see Supplementary Table S2).

The slopes of the EFR magnitude-level functions at 500, 1000 and ${2000}\,\hbox {Hz}$ (i.e. the frequencies where all listeners were considered to have “normal” audiometric thresholds) were not statistically different between the NH and the HI listeners (see Fig. 2a). In contrast, the EFR slopes at ${4}\,\hbox {kHz}$ were significantly steeper (higher values) for the HI listeners than for the NH listeners (Test statistic = 0.2963 (${95}\,\%$ CI: 0.1779, 0.4187), $p = 0.0192$). The median values of the EFR slopes for the HI listeners were ${0.40}\,\hbox {dB/dB}$, ${0.33}\,\hbox {dB/dB}$, ${0.26}\,\hbox {dB/dB}$ and ${0.57}\,\hbox {dB/dB}$ for the carrier frequencies 500, 1000, 2000 and ${4000}\,\hbox {Hz}$, respectively.

### EFR latency

It was assumed that the main EFR generator for the used modulation frequencies (80–100 Hz) was located at the level of the brainstem-midbrain^30^. In order to investigate potential large phase discontinuities, which could indicate different EFR generating sources, EFR latencies were analysed as a function of frequency and stimulation level (panels c and d in Fig. 2, respectively). A LMM revealed that there was a significant dependency of level ($\beta ={0.059}\,\hbox {ms/dB}$ SPL (${95}\,\%$ CI: 0.0566, 0.0612), t-value = 16.951, df = 61.65, $p < 2\mathrm {e}^{-16}$), but not of frequency ($p > 0.05$). A similar phase analysis using the AN model indicated that such level dependency could be of a peripheral origin (see the Discussion section for further details). EFR latencies are shown as an increment ($\Delta$ latency) with respect to the shortest measured latency. This is because the absolute latency can be misleading due to phase ambiguities in steady-state responses^38^, as the number of preceding full cycles cannot be resolved with the recording method used here. EFR $\Delta$ latencies ranged from about 3 to ${6}\,{\hbox {ms}}$.

### EFR test–retest repeatability

**Figure 4:**
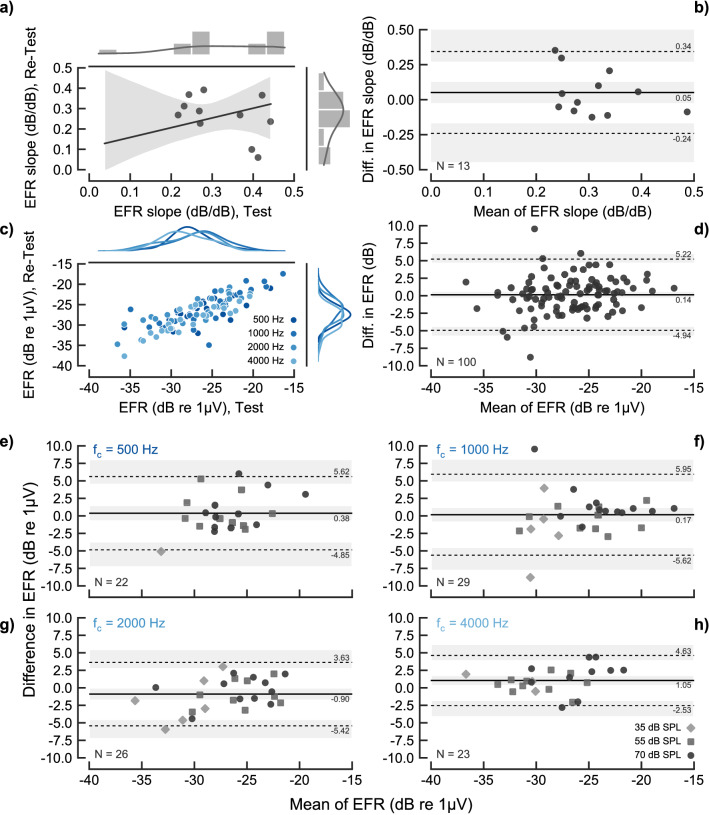
Test–retest repeatability analysis of the EFR magnitudes and the EFR slopes in the NH listeners. Panel a) show the relation between the test and retest data of the EFR slope. Top and right margins show a histogram and kernel density function of the data. The black-solid line and grey area in the main plot show the fit of a linear regression model and its ${95}\,\%$ CI. Panel (**b**) shows the difference of the test–retest EFR slopes as a function of the test–retest mean (a Bland–Altman plot). The solid-black horizontal line show the mean of the differences. The dashed-grey horizontal lines show the upper and lower LoA. The corresponding values are indicated in the right side of each line, while the grey semitransparent bands show the ${95}\,\%$ CI for each line. Panel (**c**) show the relation between the test and retest EFR magnitudes pooled across carrier frequency and level. EFR values for each carrier frequency are indicated as different intensities of blue colour. Top and right margins show kernel density functions of the distribution of the data. Panel (**d**) shows a Bland–Altman plot of the EFR test–retest data pooled across frequencies and levels. Panel (**e**–**h**) show Bland–Altman plots of the EFR test–retest data for the carrier frequencies of ${500}\,\hbox {Hz}$, ${1}\,\hbox {kHz}$, ${2}\,\hbox {kHz}$ and ${4}\,\hbox {kHz}$, respectively. Different markers indicate the stimulation level of each EFR data point, as shown in the legend in panel h). Only statistically significant EFRs were considered in the repeatability analysis, indicated by the N value in the bottom-left part of each Bland–Altman plot.

Figure 4 shows the main results of the test–retest repeatability analysis of individual EFR magnitudes (panels c–h) and EFR slopes (panels a and b). The test–retest relation of the EFR slopes (panel a) led to an ICC coefficient of 0.341 (− 0.22, 0.74), indicating a “poor” correlation. Panel b) shows a Bland–Altman plot of the same data. The solid black horizontal line indicate the mean of the differences and the grey band surrounding it indicate its ${95}\,\%$ CI. The mean of the slope difference was ${0.05}\,\hbox {dB/dB}$, indicating no systematic bias. The dashed lines show the upper and lower LoA, amounting ${0.34}\,\hbox {dB/dB}$ and ${-0.24}\,\hbox {dB/dB}$, respectively, with its ${95}\,\%$ CI depicted as grey surrounding areas. This indicates also the poor repeatability of the EFR slopes, as repeating a measurement of the EFR slope (using only two or three data points, see Methods) can lead to mean variations as large as about ± ${0.3}\,\hbox {dB/dB}$. Panel c shows the relation between individual EFR test and retest magnitudes, agnostic to carrier frequency and stimulation level. Carrier frequencies are indicated as different intensities of blue colour. The top and right margins show the kernel density function of the distributions of each frequency, showing the absence of a frequency-dependent structure in the data. The ICC coefficient was 0.793 (0.71, 0.86), indicating a “good” correlation. Panel d) shows the corresponding Bland–Altman analysis. The mean of the difference in EFR magnitudes (${0.14}\,\hbox {dB}$) did not show a systematic bias, and the LoAs indicated that the EFR test–retest repeatability was within about ± ${5}\,\hbox {dB}$. The analysis performed by segmenting the data by carrier frequency (panels e–h) showed a similar EFR test–retest repeatability for all carrier frequencies, with a slightly lower LoA for the ${4}\,\hbox {kHz}$ carrier. The ICC coefficients for each carrier frequency were: 0.679 (${95}\,\%$ CI: 0.38, 0.85) at ${500}\,\hbox {Hz}$, 0.751 (${95}\,\%$ CI: 0.54, 0.87) at ${1}\,\hbox {kHz}$, 0.817 (${95}\,\%$ CI: 0.64, 0.91) at ${2}\,\hbox {kHz}$ and 0.849 (${95}\,\%$ CI: 0.68, 0.93) at ${4}\,\hbox {kHz}$; interpreted as “good” repeatability for all frequencies except at ${500}\,\hbox {Hz}$, which is “moderate”. The repeatability results obtained in this study were similar to those presented in previous studies^67,76,77^, even though the EEG recording systems, stimuli and listeners differed across studies. The test–retest repeatability analysis was performed considering only statistically significant EFR data points. Non-significant data points were treated as missing data. The recording success rate, defined as the percentage of significant responses from the total number of recordings, was of ${63.6}\,\%$ for the test EFRs, ${77.6}\,\%$ for the retest EFRs and ${65.4}\,\%$ for all the recording pooled together.

### AN model simulations

**Figure 5:**
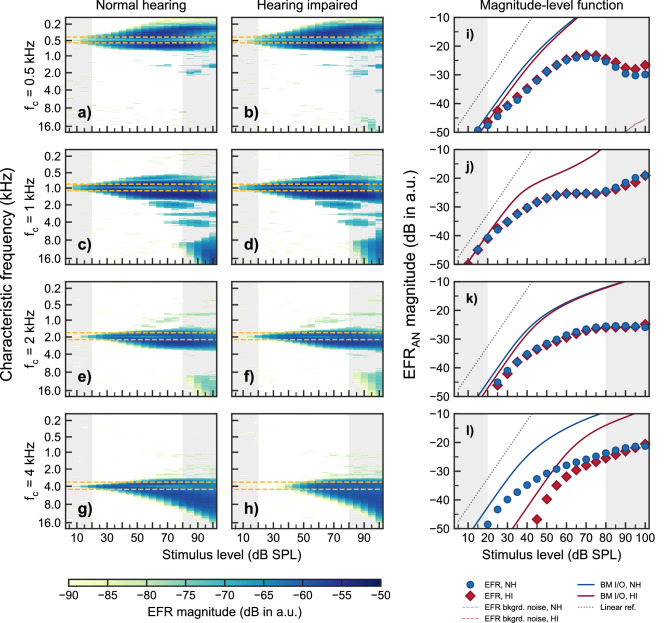
Simulated EFRs at the level of the AN ($\mathrm {{EFR}_{{AN}}}$) obtained with four simultaneously presented SAM tones as the ones used in the experiments. Panels (**a**–**h**) show $\mathrm {{EFR}_{{AN}}}$ magnitudes as a function of stimulus levels and CF for each of the four carrier frequencies (rows). The colour gradient indicate significant $\mathrm {{EFR}_{{AN}}}$ magnitudes. The horizontal orange dashed lines indicate the on-CF range as defined in^15^. The vertical grey shaded areas show input level ranges outside the level ranges considered in the experiments. The two leftmost columns show simulated $\mathrm {{EFR}_{{AN}}}$ for the mean audiogram of the NH (left) and HI listeners (middle), respectively. Panels (**i**–**j**) in the rightmost column show the corresponding $\mathrm {{EFR}_{{AN}}}$ magnitude-level functions obtained by summing up all AN activity across CF. Blue circles show NH simulations and red diamonds represent HI simulations. The thin blue and red dotted lines represent noise floor estimates (only visible in panels (**i**,**j**). The blue and red solid lines show BM I/O functions from the BM output of the model for NH and HI, respectively. The black dotted lines show a linear reference. A combination of $\frac{2}{3}$ of OHC dysfunction and $\frac{1}{3}$ of IHC dysfunction^5,79^ was assumed to adjust the AN model parameters to account for the mean audiogram values in each listener’s group.

Since BM compression represents a frequency-specific (on-CF) phenomenon^15^, a model of the AN was used to investigate the peripheral contributions to the EFR from each CF. Figure 5 shows simulated neural activity derived from the AN model by^50^ in response to the same four SAM tones (rows) as considered in the experimental recordings. Simulated envelope-based AN activity (for details regarding the calculation of $\mathrm {{EFR}_{{AN}}}$ from the AN model output, see the Methods section), both for NH and HI are presented as a function of CF and stimulus level, as well as a summed across CFs. The AN responses for the carrier frequencies 500, 1000 and ${2000}\,\hbox {Hz}$ were similar for the NH and the HI simulations (panels a-f), resulting in almost identical $\mathrm {{EFR}_{{AN}}}$ magnitude-level functions (panels i-k). At ${4}\,\hbox {kHz}$, $\mathrm {{EFR}_{{AN}}}$ magnitudes for the HI simulations (panel h and red diamonds in panel l) were not statistically significant from the noise floor at low stimulus intensities, consistent with a threshold elevation. Here, at threshold (i.e., at about 30–40 dB SPL), AN neurons tuned to a broader range of CFs responded phase-locked to the modulation frequency compared to the very narrow range of CFs in the case of the NH simulations (panel g and blue function in panel l), that showed a threshold at about 0 dB SPL. This is consistent with the broadening of frequency tuning observed in AN neurons in cochlear regions with OHC dysfunction^78^. Thus, hair-cell dysfunction led to abnormal $\mathrm {{EFR}_{{AN}}}$ magnitude-level functions, with non-significant responses at low input levels, followed by a steeper (less compressive) growth function at medium input levels that converged towards the compressive growth observed in the NH case (blue circles) at higher input levels.

The broadening of the range of contributing AN neurons with increasing stimulus level was found for all carrier frequencies (panels a–h). For each carrier frequency, the AN activity is limited to a narrow on-CF region at low stimulus levels (indicated by the horizontal orange-dashed lines in panels a–h). With increasing stimulus level, the range of AN activity broadens towards off-CF regions due to the recruitment of neurons tuned to higher CFs. This broadening is continuous for the ${4}\,\hbox {kHz}$ carrier whereas there is a saturation in the $\mathrm {{EFR}_{{AN}}}$ magnitude-level functions (panels i–k) obtained for the three lower-frequency carriers due to an interference between the neural activity of a higher frequency carrier onto a lower frequency carrier.

Solid blue and red lines in panels i–l in Fig. 5 show the growth of the BM output in the model for NH and HI, respectively, for each carrier frequency. The BM magnitude-level functions show a linear growth at low stimulus levels that bends towards a compressive growth at medium-to-high levels. This is less clear at ${500}\,\hbox {Hz}$ because the BM gain in the model is lower at this frequency^80^. With a mild hearing impairment (panel l) the BM output shows reduced sensitivity at low stimulus levels but residual compression at higher levels. The compression values estimated from the output of the BM in the model are relatively similar to the compression values estimated from simulated $\mathrm {{EFR}_{{AN}}}$ magnitude-level functions. The same piecewise linear function with two segments as used in the experimental EFR data was used here for the model simulations. The compression values from the simulated NH BM output amounted 0.62, 0.33, 0.26 and ${0.27}\,\hbox {dB/dB}$ at the four carrier frequencies, respectively; versus 0.58, 0.34, 0.39 and ${0.23}\,\hbox {dB/dB}$ at the same frequencies for the $\mathrm {{EFR}_{{AN}}}$ magnitude-level functions. In the case of simulated HI, BM compression values were 0.66, 0.33, 0.27 and ${0.32}\,\hbox {dB/dB}$ versus 0.59, 0.32, 0.36 and ${0.25}\,\hbox {dB/dB}$ for the $\mathrm {{EFRs}_{{AN}}}$. Despite the similarity, one should bear in mind that the BM output for a given carrier frequency reflects the response of the cochlear segment tuned to that specific frequency, i.e., an on-CF response; whereas $\mathrm {{EFR}_{{AN}}}$ magnitude-level functions result from the addition of any CF responding to the modulation frequency, as shown in colour gradient in panels a-h in Fig. 5. A comparison between on- and off-CF contributions to the $\mathrm {{EFR}_{{AN}}}$ is discussed below.

## Discussion

### Compression estimates based on EFR magnitude-level functions

The slopes of the experimental EFR magnitude-level function for the NH listeners varied between 0.2 and ${0.35}\,{\hbox {dB/dB}}$. They were not statistically different from the slopes at the non-impaired frequencies (500, 1000 and ${2000}\,\hbox {Hz}$) for the HI listeners (see Fig. 2a). On a group average, these values are similar to BM compression estimates obtained using direct invasive methods in healthy non-human animal models, and with non-invasive physiological and behavioural compression estimates in NH humans. Compression values estimated from the slope of BM velocity-intensity I/O functions in chinchillas at medium-to-high stimulation levels (40–90 dB SPL) varied between 0.2 and ${0.5}\,\hbox {dB/dB}$^15^. Group-averaged psychoacoustical compression estimates in NH humans were found to be between 0.15 to ${0.35}\,\hbox {dB/dB}$ (e.g.,^19,20,81–85^). Compression estimates using group-averaged DPOAE magnitude-level functions were shown to be of about ${0.2}\,\hbox {dB/dB}$ in NH listeners at moderate stimulus levels (50 to 70 dB SPL) (e.g.,^25,26,28^).

Some of the characteristics in the data of the HI listeners seemed also consistent with the results in literature obtained using other cochlear compression estimates. The slopes at ${4}\,\hbox {kHz}$ (where the HI listeners showed a mild hearing loss) were significantly steeper than the corresponding slopes for the NH listeners (Fig. 2a). The steeper growth function in the HI listeners is consistent with the concept of reduced BM compression observed or estimated with other methods (e.g.,^18–20,28,86^). In addition, the increase of the lowest stimulus level at which an EFR could be measured in the HI listeners is consistent with the corresponding increased pure-tone threshold at that frequency (see also^39,87–92^). Thus, based on the group-averaged numerical values, the similarity of the compression estimates obtained across the different methods, including the EFR, may suggest that similar aspects of peripheral auditory processing are reflected in the different measures.

However, while the slope of the compressive part of the respective level-growth functions is similar across methods, the overall shape of the magnitude-level functions differs. For example, while a change in the slope of the magnitude-level functions (often referred to as a “break-point” or “knee point”) can be identified both in the EFR results as well as in the behavioural measures, there are substantial differences. In fact, in the behavioural studies, a break-point has typically been estimated at stimulus levels at or below about ${45}\,\hbox {dB}$ SPL (e.g.,^12,20,81,84,93^), whereby the slope of the estimated BM I/O function has been usually approximates linear growth at lower levels. The slope beyond the break-point, at medium-to-high levels, commonly grows compressively in NH listeners. In contrast, for the EFR magnitude-level functions obtained in the present study, no linear growth was found, in any of the listeners at any frequency, at the lowest levels considered. While this characteristic of the EFR magnitude-level seems inconsistent with the behaviourally estimated BM I/O functions, it is not inconsistent with data from non-human animal recordings. These recordings show that linearised BM growth can occur at stimulus levels below 20 dB SPL (see Fig. 3 in^15^), which were input levels not tested in the present study. In addition, the simulated $\mathrm {{EFR}_{{AN}}}$ magnitude-level functions showed growth ratios close to ${1}\,\hbox {dB/dB}$ only at very low stimulus levels below ${30}\,\hbox {dB}$ SPL (panels i–l in Fig. 5), impossible to see from the recorded data. Furthermore, the experimental EFR magnitude-level functions showed a break-point at about 50–65 dB SPL (see Fig 2e); i.e., at higher levels than in the behavioural studies. This break-point actually reflected the transition between the compressive growth at low-medium stimulus levels and the level region where the EFR magnitudes saturated (at 500, 1000 and ${2000}\,\hbox {Hz}$), consistent with a previous study^42^. Thus, the characteristic of the EFR magnitude-level function, including its compressive behaviour, do not seem to reflect the same processes that underlie the behaviourally estimated BM I/O function. The same may hold in relation to the level-growth functions obtained with non-invasive physiological methods such as DPOAEs as well as the invasive (non-human) measures, as further outlined below.

It was assumed that EFR elicited by modulation frequencies of 80-100 Hz were mainly generated at the level of the brainstem-midbrain^30^. In order to investigate the effect of stimulus level on the EFR generation, EFR phases converted to latency-level functions^94^ were analysed (Fig. 2d). A LMM indicated a significant effect of level on EFR latency with a growth of ${0.059}\,\hbox {ms/dB}$, but no effect of frequency. Such increase of EFR latency with increasing stimulus level is in agreement with the results in^95^, but in disagreement with another study from the same group^94^, although the later study tested only two stimulus levels. The average change of latency amounted to ${3.55}\,\hbox {ms}$ (from 20–80 dB SPL), similar to about ${3.8}\,\hbox {ms}$ (from 40 to 80 dB SPL) in^95^ and ${2.4}\,\hbox {ms}$ (from 35-to 75 dB SPL) in^94^, which may indicate a similar generator across level. To investigate the potential peripheral origin of the level dependency, phase-level slopes were estimated from the simulated $\mathrm {{EFR}_{{AN}}}$ functions and subtracted to the experimental EFR phase-level functions. Applying the same LMM, stimulus level became statistically non-significant ($\beta ={0.005}\,\hbox {ms/dB}$ SPL (${95}\,\%$ CI: 0.0017, 0.0078), t-value=1.105, df=43.74, $p = 0.2751$). Thus, it seems that this small increase in latency with increasing level is of peripheral origin.

### Comparison of compression estimates using EFRs and DPOAEs

**Figure 6:**
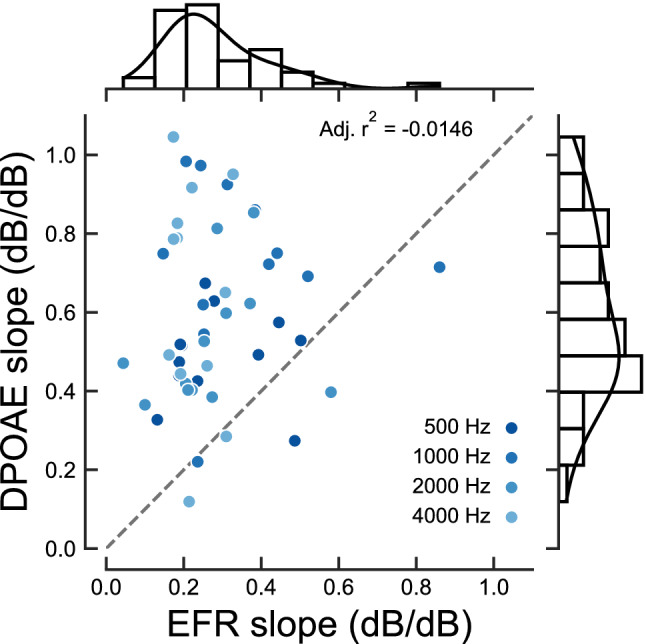
Relation between EFR slopes and DPOAE slopes in the same NH listeners. Frequencies are indicated as different intensities of blue colour. The dashed dark-grey line show a 1:1 ratio. Top and right marginal histograms and kernel density functions show the EFR and DPOAE slopes distributions (pooled across frequency), respectively.

Figure 6 shows the relation between estimated EFR and DPOAE slopes in the same NH listeners. Correlation analysis using Shepherd’s pi correlation^96^ resulted in a correlation coefficient of $r=0.1689$ (${95}\,\%$ CI: − 0.12, 0.43, adj. $r^2 = -\,0.0146$, $p = 0.2618$), indicating a poor relation. In general, DPOAE compression estimates were higher (less compressive) and more variable than EFR compression estimates (panels a and b in Fig. 2 and marginal histograms in Fig. 6). The lack of correspondence between different methods proposed to estimate cochlear compression is not new. One study showed that cochlear compression estimates using DPOAEs were not correlated with behavioural compression estimates in the same individual listeners^97^. Another study showed a correlation only at ${4}\,{\hbox {kHz}}$ but not at lower frequencies^82^, and the correlation did not become stronger when efforts were made to reduce the DPOAE variability^98^.

EFR compression estimates (median values between 0.2 and ${0.35}\,\hbox {dB/dB}$) were more in agreement with cochlear compression estimates derived using other methods reported in literature than DPOAE compression estimates (median values between 0.5 and ${0.74}\,\hbox {dB/dB}$). At a first glance, this could indicate that peripheral compression is better estimated with EFR slopes than with DPOAE slopes, or at least that different underlying mechanisms are being captured by the different methods. The AN modelling analysis, where such underlying mechanisms can be elucidated, suggested that EFR slopes do not reflect cochlear compression (see below in the Discussion). The DPOAE slopes reported here are consistent with slopes reported in some studies^28,99^, but are much larger (less compressive) than in other studies^25,26^. Different experimental parameters such as primary levels and primary frequency ratios, or physiological aspects like OHC status could explain the discrepancy between studies and the large variability observed across listeners. For example, it has been suggested that the place on the BM where the second primary ($f_{2}$) travelling wave peaks, which affects the main DPOAE generation site, depends on the used primary level paradigm^100^. Biophysical cochlear models could be used to determine the optimal set of parameters needed to estimate cochlear compression with OAE, but this is beyond the scope of the current study.

### EFR test–retest repeatability

The repeatability analysis of individual EFR recordings showed a “good”^68^ test–retest repeatability based on the ICC method. The Bland–Altman method indicated that EFRs can fluctuate between about ± ${5}\,\hbox {dB}$ from session to session. However, the test–retest repeatability of the EFR slope between 35 and ${55}\,\hbox {dB}$ SPL (for 500, 1000 and ${2000}\,\hbox {Hz}$) and based on the three retest data points at ${4}\,\hbox {kHz}$ was “poor”, probably due to error propagation, as more than one EFR data point is used for estimating the slope. This is problematic for a potential clinical use of the EFR slope. The repeatability results of individual EFRs were consistent with other reported analyses. The repeatability of individual EFR magnitudes was reported to be corresponding to ICC = 0.93 (“excellent”) and ICC = 0.71 (“good”) for fully modulated ($\mathrm {m}=100 \%$) and shallow modulated ($\mathrm {m}=50 \%$) EFRs, respectively^67^, leaving the repeatability coefficient found in the present study with modulation depths of $\mathrm {m}=85 \%$ in between the two other reported studies (ICC = 0.792). Similarly, the Pearson correlation was reported to be $r=0.91$ in another study that used single carriers modulated at $\mathrm {m}=100 \%$ presented at ${50}\,\hbox {dB}$ HL^77^. Moreover, the LoAs of EFRs elicited also by a single carrier frequency modulated at $\mathrm {m}=100 \%$ presented at ${50}\,\hbox {dB}$ HL were found to be of about ± ${40}\,\%$ of the mean linear EFR amplitude, corresponding to about ± ${3}\,\hbox {dB}$^76^, in comparison to about ± ${5}\,\hbox {dB}$ in the present study.

### On- versus off-CF contributions to EFR compression estimates

**Figure 7:**
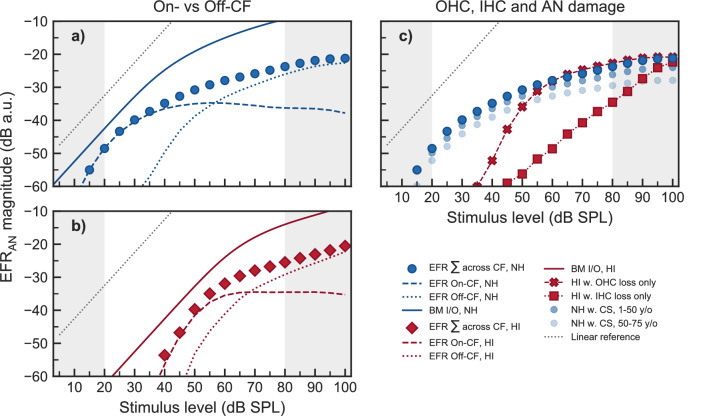
Analysis of on- vs off-CF contributions and OHC vs IHC dysfunction on the simulated $\mathrm {{EFR}_{{AN}}}$ magnitude-level functions from the 4 kHz component. Panel (**a**) shows NH simulated $\mathrm {{EFR}_{{AN}}}$ magnitude-level function after summing contributions across CF (circles), the contribution to $\mathrm {{EFR}_{{AN}}}$ from neurons in the on-CF range (dashed line) and the contributions from off-CF neurons (dotted line). The solid line shows the growth of the BM output. Panel (**b**) shows the same as panel a but assuming hearing impairment with a combinations of $\frac{2}{3}$ of OHC dysfunction and $\frac{1}{3}$ of IHC dysfunction. Panel (**c**) shows the same simulated HI $\mathrm {{EFR}_{{AN}}}$ magnitude-level functions as in panel (**b**) (summed across CFs) but assigning the threshold elevation to only OHC dysfunction (red crosses, dashed line) or to only IHC dysfunction (red squares, dotted line). The NH simulation is shown by blue circles as a reference. The grey-dotted line in all panels indicate a linear growth.

The compressive growth of BM I/O functions measured locally in animal models reflects on-CF responses at a narrow BM range. At off-CF places, BM I/O functions have been demonstrated to grow linearly (see Figs. 6 and 7 in^15^). Thus, in order to estimate on-CF (i.e., place- or frequency-specific) compression using EFRs (or any other method), the response needs to be dominated by such on-CF processing. At low intensities, a narrow-band stimulus (e.g., a SAM tone) excites a narrow region of the BM and a small part the AN population. Thus, the EFR responses are likely to be dominated by the activity of a small population of neurons tuned to the centre frequency of the stimulus. However, at medium and high stimulus levels, the excitation pattern of the BM broadens and a larger population of AN neurons tuned to frequencies remote from the centre frequency contribute to the gross activity. Indeed, based on simulations of neural activity at the level of the AN using the model by^72,101^, it was shown that responses to a single SAM tone presented at medium-to-high stimulus levels are dominated by contributions from off-CF HSR fibres^41^. This is despite the fact that the maximum of the BM excitation is located at on-CF.

When presenting more than one SAM tone simultaneously, the presence of a SAM tone of higher carrier frequency prevented a SAM tone at a lower frequency to recruit AN neurons tuned to higher CFs (Fig. 5, see panels a-f). This was not the case for the ${4}\,\hbox {kHz}$ component (panels g and h), where off-CF basal neurons could be recruited without interference from another SAM tone. This is supported by findings from invasive recordings in non-human animals showing that AN fibres can follow the periodicity of a high level tone with frequency energy below the CF of the fibre (e.g.,^102,103^). Also, EFRs recorded in rats have shown the effect of a second, high-frequency SAM carrier onto the encoding of a low-frequency SAM carrier^104^. Consistent with the model simulations in the present study, the study by^104^ concluded that the presence of the second SAM tone basal to the place of the main low-frequency SAM tone caused a reduction of the EFR to the lower-frequency component due to reduced recruitment of AN fibres located basally relative to the on-CF location. Such interaction of a high-frequency SAM carrier onto the AN activity induced by a lower-frequency carrier is the reason underlying the saturation of the simulated $\mathrm {{EFR}_{{AN}}}$ magnitude-level functions above 50–60 dB SPL, as observed at 500, 1000 and ${2000}\,\hbox {Hz}$ (panels i and j in Fig. 5). At ${4}\,\hbox {kHz}$, the model shows a monotonic growth of $\mathrm {{EFR}_{{AN}}}$ magnitudes at stimulus levels above ${30}\,\hbox {dB}$ SPL in the NH simulations (panel l in Fig. 5). This is consistent with the single-slope growth function observed in the experimental EFR data (panel d in Fig. 1 and panel b in Fig. 2). This is because off-CF basal neurons could be recruited at high stimulus levels without interference, strongly contributing to the compound response. Similarly, simulated $\mathrm {{EFR}_{{AN}}}$ magnitude-level functions using a single SAM tone at ${2}\,\hbox {kHz}$ resulted also in non-saturating (monotonically increasing) growth functions (see Fig. 4a in^41^). Thus, the model offers a comprehensive explanation for the saturation of the EFR magnitude-level functions observed at 500, 1000 and ${2000}\,\hbox {Hz}$ in the recorded data (see panels a–c in Fig. 1 and Fig. 3).

The orange-dashed lines in Fig. 5 (panels a–h) indicate the limits of the on-CF range as derived from direct BM recordings in non-human mammals^15^. Within this on-CF range, the $\mathrm {{EFR}_{{AN}}}$ magnitude first increases with input level, shows a maximum at about 25-${30}\,\hbox {dB}$ above threshold, and then decreases with increasing stimulus level. This is more clearly shown in the dashed-blue function in Fig. 7a. At medium-to-high stimulus levels, the modulation (and therefore the $\mathrm {{EFR}_{{AN}}}$) was found to be more dominant at off-CFs than at on-CFs (see also panels a and b in Fig. 7). At these higher levels, many on-CF HSR fibres will saturate in rate and do not strongly encode the modulations. In contrast, AN neurons tuned to off-CFs (including HSR fibres) are still excited below saturation level, due to their lower off-CF sensitivity (as reflected in their tuning curves). Thus, the off-CF HSR neurons more robustly encode the amplitude modulations at these higher levels. This is consistent with physiological recordings from the cat AN showing that off-CF AN fibres exhibit higher synchrony to high intensity SAM tones than on-CF AN fibres^105^. Nonetheless, the $\mathrm {{EFR}_{{AN}}}$ magnitude-level function obtained after summing across CF continued to grow monotonically due to a further increase of off-CF contributions. Panels a) and b) in Fig. 7 show the overall contribution of on- and off-CFs to the compound response to the ${4}\,\hbox {kHz}$ SAM tone. The NH simulations are shown in blue and the HI simulations are indicated in red. The compressive slope estimated by fitting the piecewise function to the $\mathrm {{EFR}_{{AN}}}$ magnitude-level function (blue circles in panel a) results from the mixture of on- (dashed-blue line) and off-CF (dotted-blue line) contributions. The solid-blue line shows the BM output at ${4}\,\hbox {kHz}$, which reflects a purely on-CF response, and hence shows a compressive growth at medium-to-high stimulus level. At the same level range, the corresponding $\mathrm {{EFR}_{{AN}}}$ on-CF response (dashed-blue line) shows a decreasing function with increasing level, consistent with physiological results in non-human animals^105^. For this particular carrier frequency, the compression value from the simulated BM was ${0.27}\,\hbox {dB/dB}$, very similar to the ${0.23}\,\hbox {dB/dB}$ compression value from the total simulated $\mathrm {{EFR}_{{AN}}}$ magnitude-level function. But it was completely different from the slightly negative growth of ${-\,0.02}\,\hbox {dB/dB}$ from the on-CF $\mathrm {{EFR}_{{AN}}}$, and similar to the ${0.29}\,\hbox {dB/dB}$ growth from the off-CF $\mathrm {{EFR}_{{AN}}}$. The same effect can be seen in the HI simulations (see Fig. 7b). Therefore, while BM compression purely reflects on-CF processing, the estimates of compression obtained from EFR magnitude-level functions do not exclusively reflect on-CF cochlear compression, because its generation in the AN is strongly influenced by off-CF contributions, according to the model. This is conceptually consistent with the limitations of estimating place-specific cochlear dispersion using EFRs^106^.

IHC dysfunction (but intact OHC function) is considered to lead to a BM I/O function with comparable compression estimates as in the NH listeners (e.g.,^81^). Figure 7c shows simulations of the ${4}\,\hbox {kHz}$ $\mathrm {{EFR}_{{AN}}}$ magnitude-level function when accounting for the mild threshold elevation with only OHC dysfunction (red crosses, dashed line) or with only IHC dysfunction (red squares, dotted line). Even though both types of hair-cell dysfunction led to a similar threshold elevation of 30-40 dB SPL, the growth function showed different shapes. In the case of only OHC dysfunction, $\mathrm {{EFRs}_{{AN}}}$ grew steeply just above threshold and became as compressive as in the NH case (blue circles) at levels beyond about ${60}\,\hbox {dB}$ SPL. This steeply growing part, dominated by on-CF processing (see dashed-red line in panel b), resulted as a consequence of the loss of local on-CF gain due to simulated OHC dysfunction. The compressive growth that follows it at levels beyond ${60}\,\hbox {dB}$ SPL resulted from the off-CF processing of the ${4}\,\hbox {kHz}$ SAM tone by basally tuned AN neurons (i.e., off-CF contributions). These neurons are not affected by OHC dysfunction because they are excited through the tails of their tuning curve^78^. In the case of only IHC dysfunction, processing of both on- and off-CF AN neurons was affected, as their whole tuning curve shifts towards higher levels^78^. Even though cochlear gain was not reduced (see the sharply tuned response in Supplementary Fig. S5h) and residual BM compression was “normal” (see the solid-red line in panel b), this resulted in an $\mathrm {{EFRs}_{{AN}}}$ function increasing monotonically with a mildly compressive single slope (red squares, dotted line in Fig. 7c). Therefore, the reduction of BM compression due to OHC dysfunction cannot be extracted from the simulated $\mathrm {{EFRs}_{{AN}}}$ magnitude-level functions because of the dominance of off-CF contributions at higher stimulus levels.

It was assumed in the hypothesis that all possible compressive sources beyond the BM were similarly affected by a reduction of BM compression due to OHC dysfunction. This assumption could be compromised by the effect of CS on the slope of EFR magnitude-level functions. In mice, in contrast to noise-induced CS^107^, age-induced CS produce a wide-spread loss of AN synapses along the tonotopical axis^48^. Changes of EFR slope were reported in^48^, but only for mice older than 64 weeks which also presented signs of OHC dysfunction. As shown in Fig. 7c, on-CF OHC dysfunction can lead to changes in the slope and shape of EFR magnitude-level functions. In ageing human temporal bones, CS was found to be wide-spread too^49^. This may lead to an overall reduction of EFR magnitudes^108,109^ but may not produce a significant change in EFR slopes^41^. To confirm this, we simulated $\mathrm {{EFRs}_{{AN}}}$ magnitude-level functions imposing synaptic losses (agnostic to AN fibre type) according to the age groups of 1-50 and 50-75 years old in^49^ (Fig. 7c), that could account for the about 30 years difference between our NH and HI listeners. The estimated $\mathrm {{EFRs}_{{AN}}}$ slopes for the 1-50 and 50-75 years old groups were ${0.23}\,\hbox {dB/dB}$ and ${0.18}\,\hbox {dB/dB}$, respectively; in contrast to ${0.23}\,\hbox {dB/dB}$ in the healthy model. We consider that this subtle change in slope in the older age group does not invalidate our assumption. In addition, one could argue that a predominant loss of low-SR fibres could produce a change in slope by reducing more the EFR magnitude to higher stimulus levels^110^. However, a similar modelling analysis showed that the impact of selective medium- and low-SR loss on EFRs was marginal^41^. In addition, recent physiological results from single-unit bushy cells in the cochlear nucleus (CN) predominantly innervated by medium- and low-SR AN fibres showed that these neurons respond only to the onsets, and not to the steady-state when stimulated by train pulses^111^. This suggests that these neurons may not contribute to responses such as the EFR or the frequency following response (FFR).

The assumption of reflecting frequency-specific local compression might also be challenged in the case of other measurement paradigms, such as those based on DPOAEs or psychoacoustical masking paradigms. The slope of DPOAE magnitudes-level functions was proposed to be used as an estimate of local BM compression^25^. The distortion source of the emission is usually simplified as a single source located at the peak of the travelling wave envelope (at on-CF) of the $f_2$ primary, although many distortion sources might be induced in the region where the travelling waves of the two primaries overlap^112^. The extent of potential off-CF contributions to the non-linear component of the DPOAE at high stimulus levels is not yet fully understood^113^, which could be a potential confound in the estimate of BM compression. Regarding behaviourally obtained estimates of BM I/O functions, on- and off-CF maskers have been used in a forward-masking paradigm. Using high-level off-CF maskers may thereby lead to an overestimation of compression by as much as a factor of 2^114^. Furthermore, physiological recordings in non-human mammals demonstrated that the amount of forward masking in the AN is not large enough to account for the behavioural forward masking, whereas physiological masking at the level of the inferior colliculus seems to be reflecting behavioural patterns^115^. Thus, behaviourally estimated BM I/O functions derived from forward masking paradigms may reflect mechanisms beyond cochlear processing. A modelling analysis as the one provided in the current study for evaluating the potential peripheral neural generators contributing to the EFRs might be useful also for exploring the contributing factors underlying level-growth functions obtained with, e.g., DPOAEs and psychoacoustical masking measures that have been used to estimate cochlear compression. For instance, cochlear transmission-line models (e.g.,^116,117^) could be used to validate methods based on DPOAEs (or other oto-acoustic emissions), and an integration of the current AN model^50^ with signal detection theory methods (e.g.,^118,119^) could be applied to validate behavioural estimates of compression.

## Conclusion

The recorded EFR magnitude-level functions showed a compressive growth with slopes comparable to compression estimates using direct physiological recording in non-human mammals, and group-averaged results from psychoacoustical methods as well as DPOAE magnitude-level functions. In the case of a mild threshold elevation, the estimated slopes were higher than in the case of NH, also consistent with the interpretation of a less compressive response due to a reduction of cochlear gain. The slope of DPOAE magnitude-level function was used as another physiological estimate of cochlear compression in the same NH listeners. Compression estimates from DPOAEs were found to be larger (less compressive) than EFR-based estimates, and there was no correlation between the two metrics. This could indicate different potential underlying mechanisms. Moreover, a test–retest analysis revealed that, while the individual EFR recordings showed good repeatability, the repeatability of the slope estimated from EFR magnitude-level functions was poor due to error propagation.

A computer model of the AN was used to simulate $\mathrm {{EFRs}_{{AN}}}$ magnitude-level functions. The model was able to correctly account for the saturation of the EFRs above ${60}\,\hbox {dB}$ SPL observed at the carrier frequencies of 0.5, 1 and ${2}\,\hbox {kHz}$, and it was able to account for the single-slope monotonic growth at the highest carrier frequency of ${4}\,\hbox {kHz}$. A model analysis revealed that the compression values estimated from the simulated $\mathrm {{EFR}_{{AN}}}$ magnitude-level functions, obtained by summing up contributions from responding neurons across CF, was similar to the compression values estimated from the growth of the BM output, obtained at the on-CF cochlear segment. However, the model suggested that $\mathrm {{EFR}_{{AN}}}$ magnitudes at medium-to-high stimulus levels contained substantial off-CF contributions. According to the model, this was the underlying mechanism leading to the compressive growth of the $\mathrm {{EFR}_{{AN}}}$ magnitude-level functions. Indeed, the on-CF contribution of simulated $\mathrm {{EFR}_{{AN}}}$ magnitude-level functions decreased with increasing level, which is at odds with the increasing compressive growth in the BM. In conclusion, despite the numerical match between the compressive growth of EFR magnitude-level functions and estimates of cochlear compression using other methods, the model suggested that the compressive growth of the EFR magnitude-level functions may result from off-CF contributions, and not from the local on-CF compressive growth in the BM. This compromises the use of EFRs to estimate peripheral compression, together with the poor test–retest repeatability of the estimated slopes.

## Supplementary information

Supplementary Information.
