# Supplementary material for: On the use of envelope following responses to estimate peripheral level compression in the auditory system
Source: Sci Rep. 2021 Mar 26;11:6962. doi: 10.1038/s41598-021-85850-x (PMC7997911; doi:10.1038/s41598-021-85850-x)
Supplement: Supplementary file 1 — Supplementary Information. [file 41598_2021_85850_MOESM1_ESM.pdf]

SUPPLEMENTARY MATERIAL:

Title:

On the Use of Envelope Following Responses to Estimate Peripheral Level Compression in the Auditory System

Authors:

Gerard Encina-Llamas, Torsten Dau and Bastian Epp

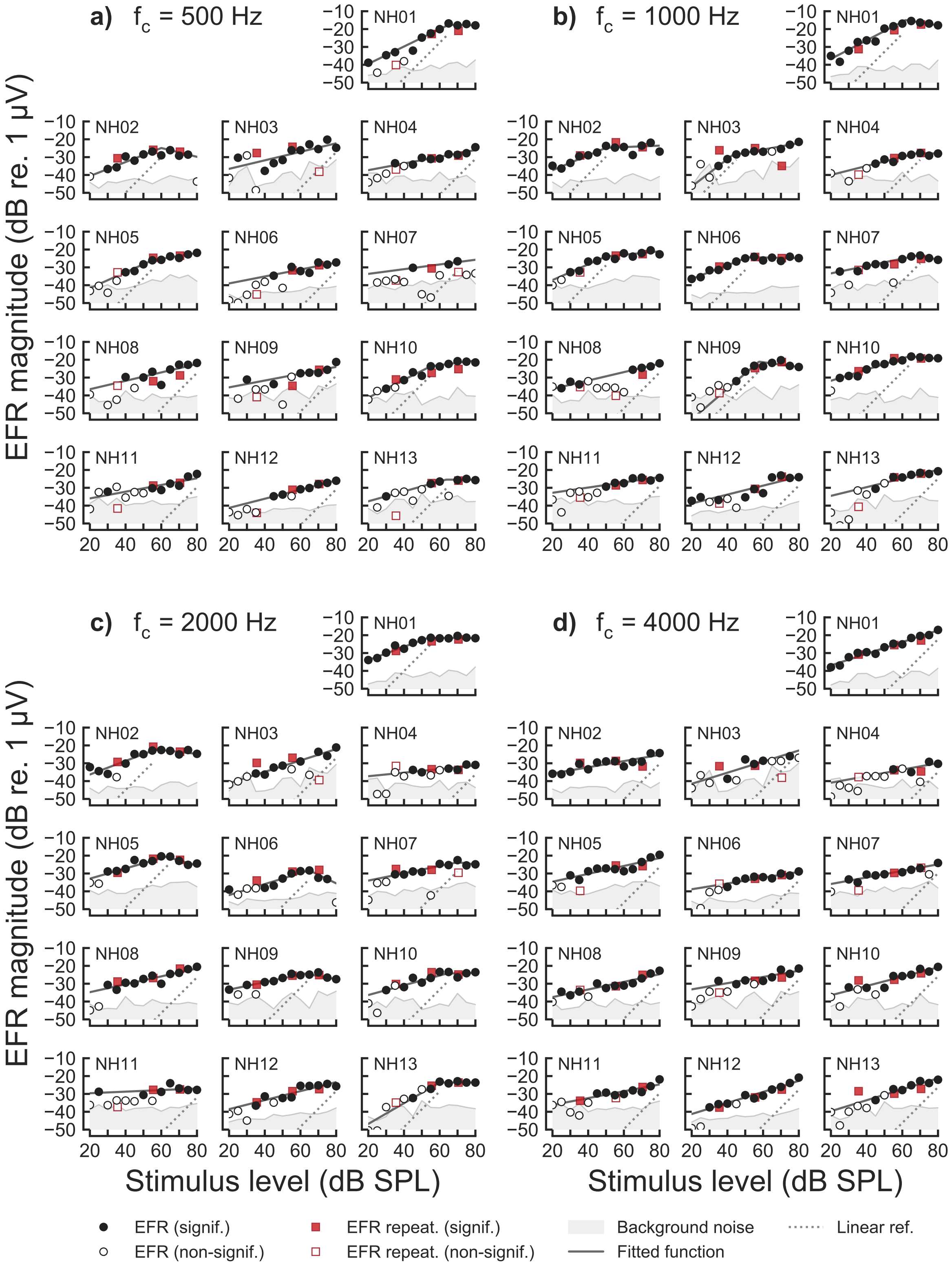

Supplementary Figure 1. EFR magnitude-level functions recorded in all NH listeners for the carrier frequencies of a) 500 Hz, b) 1000 Hz, c) 2000 Hz and d) 4000 Hz. EFR magnitudes are represented as filled symbols in the case of a statistically significant response (positive F-test), and as open symbols in the case of statistically non-significant (negative F-test) responses. Circles indicate the EFR magnitudes recorded in the first recording session and red squares represent EFR magnitudes recorded in the second recording session. EEG background noise estimates are shown as the grey shaded area. The best fitted curves are represented by the solid dark-grey line. Linear reference with slope of 1 dB/dB is indicated by the dotted line.

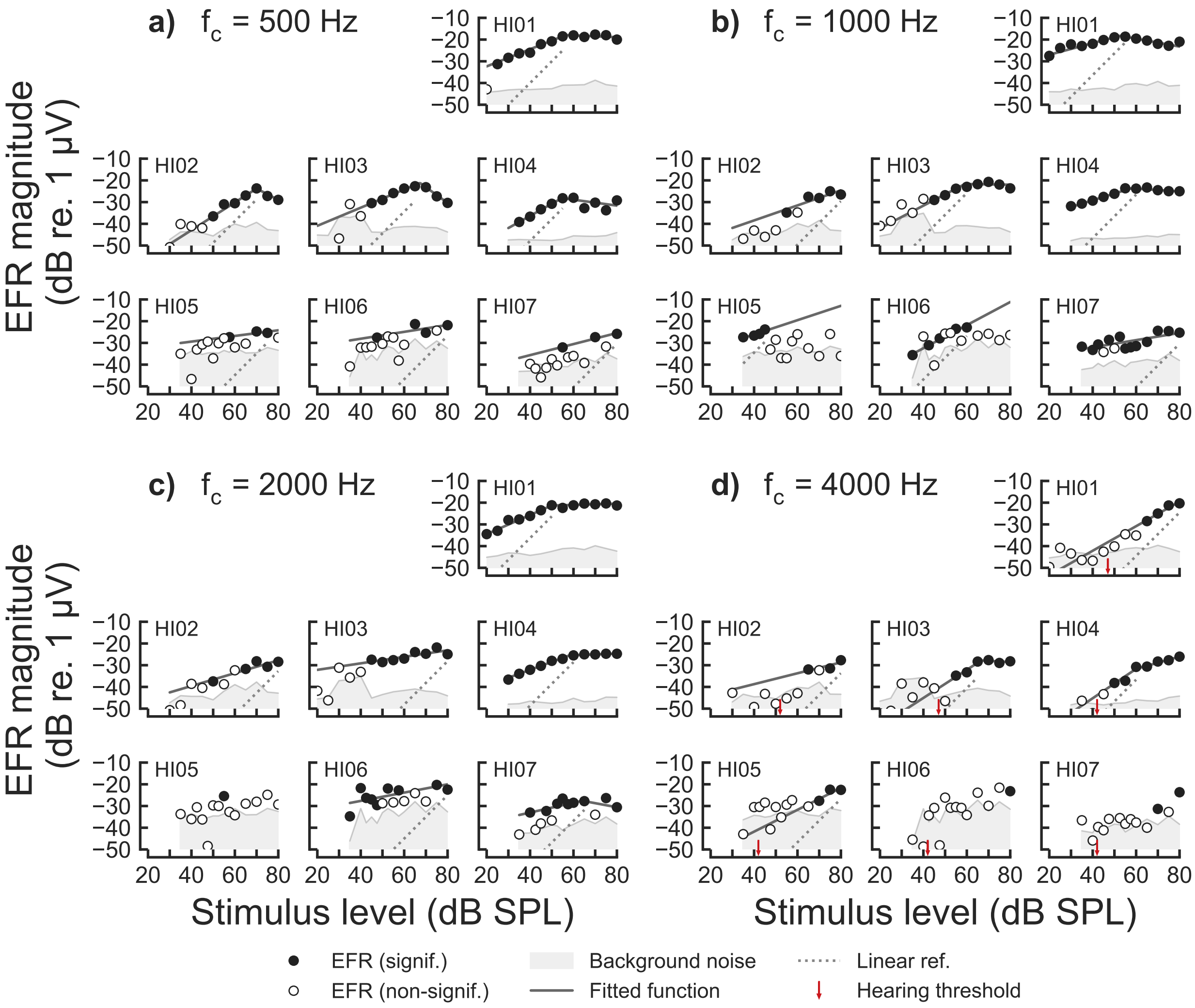

Supplementary Figure 2. EFR magnitude-level function recorded in all HI listeners for the carrier frequencies of a) 500 Hz, b) 1000 Hz, c) 2000 Hz and d) 4000 Hz. Same representation as in Fig. 1 but not including the repeatability measurements. The small red arrow in panel d) indicates the behavioural hearing threshold of the listener at 4000 Hz in dB SPL.

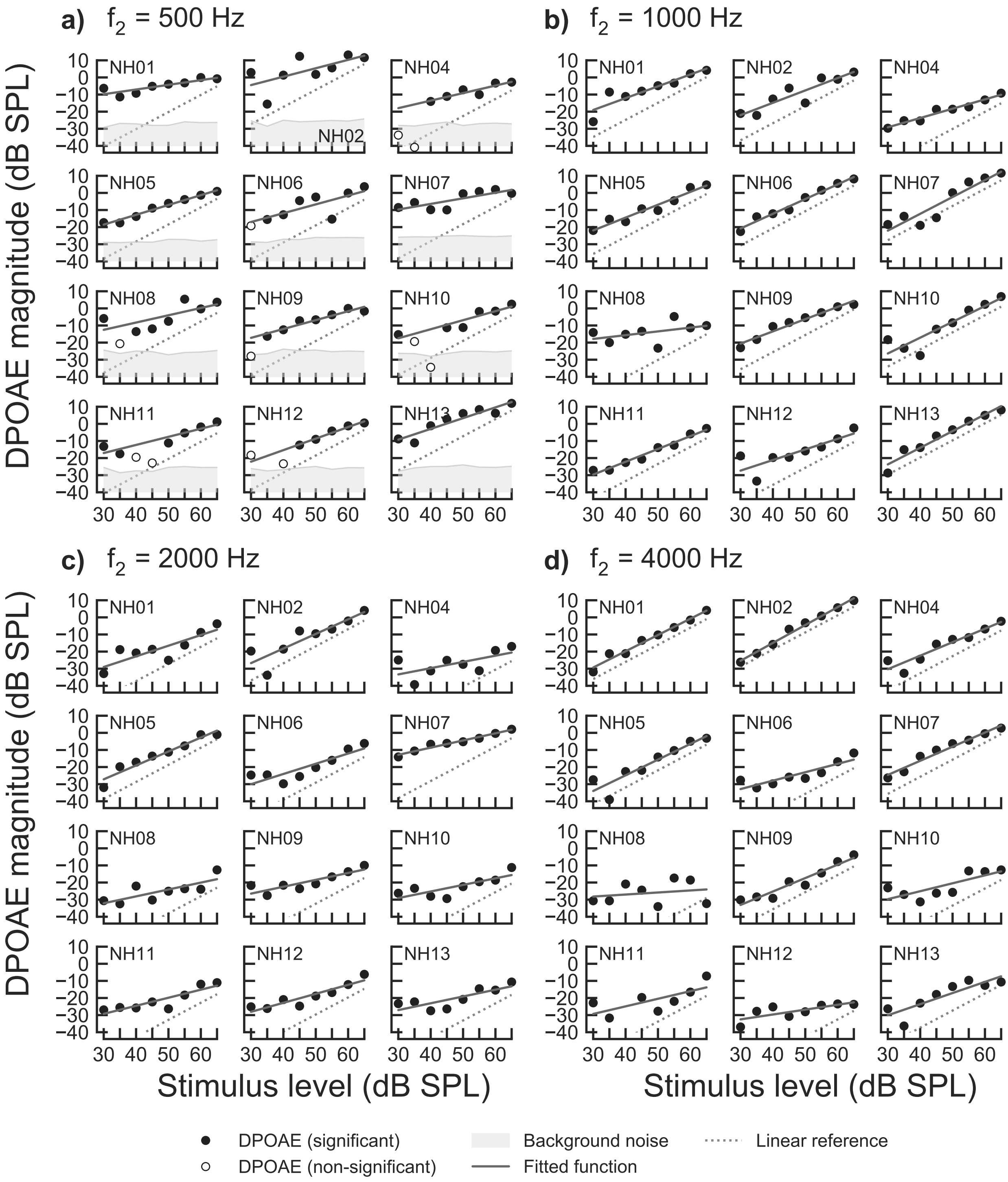

Supplementary Figure 3. DPOAE magnitude-level functions recorded in all NH listeners for the carrier frequencies of a) 500 Hz, b) 1000 Hz, c) 2000 Hz and d) 4000 Hz. DPOAE magnitudes are represented as filled symbols in the case of a statistically significant response (SNR≥10 dB), and as open symbols in the case of statistically non-significant (SNR<10 dB) responses. Background noise estimates are shown as grey shaded areas. Linear fits to the data are represented by the solid dark-grey line. Linear reference with slope of 1 dB/dB is indicated by the dotted line.

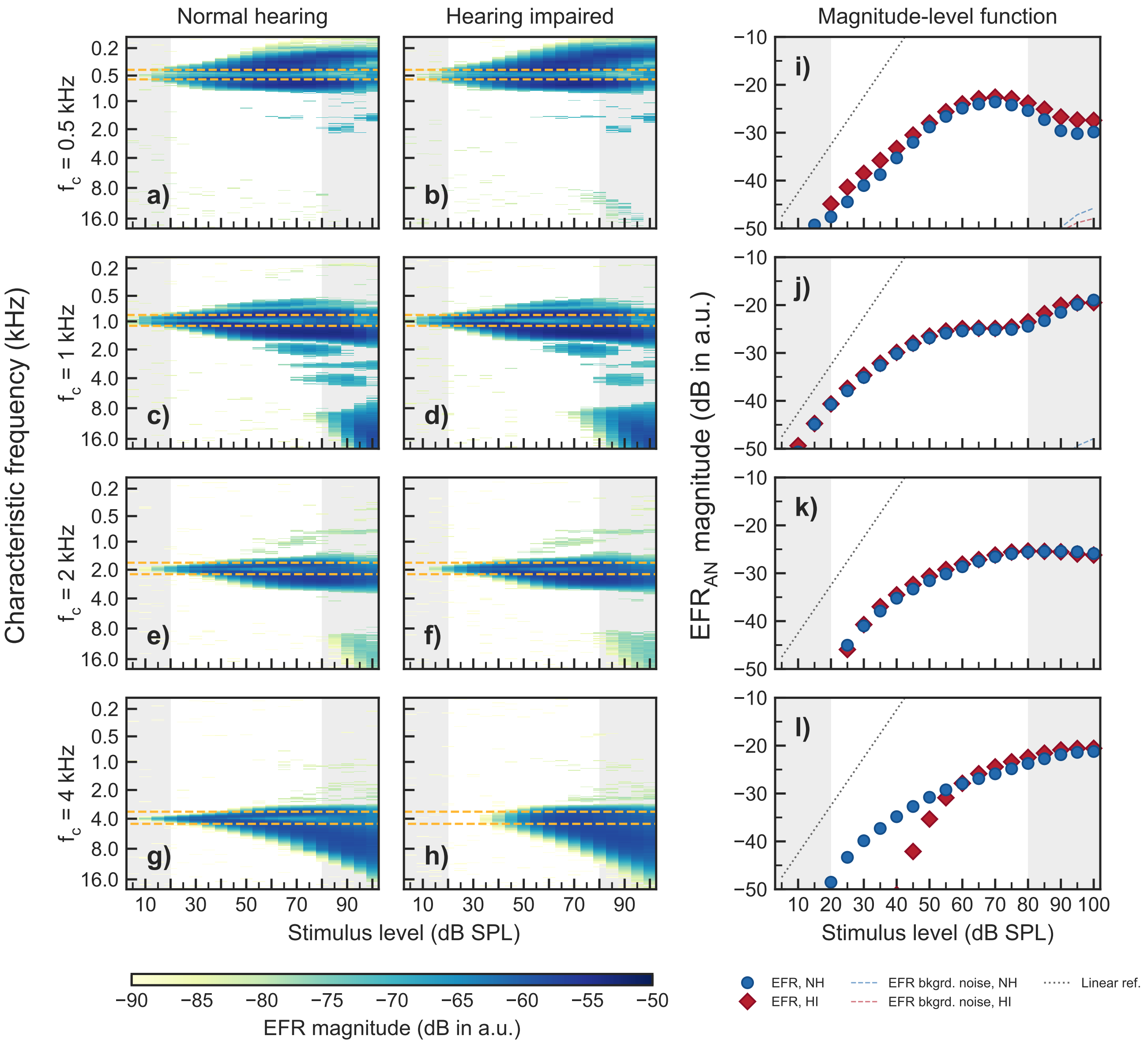

Supplementary Figure 4. Simulated EFR at the level of the AN (EFR_{AN}) obtained with four simultaneously presented SAM tones as the ones used in the experiments for NH and HI assuming only OHC dysfunction. Panels a-h show EFR_{AN} magnitudes as a function of stimulus levels for each of the four carrier frequencies (0.5, 1, 2 and 4 kHz). The colour gradient indicate significant EFR_{AN} magnitudes. The horizontal orange dashed lines indicate the on-frequency range as defined in the Results section in the article text. The vertical grey shaded areas show input level ranges outside the level ranges considered in the experiments. The two leftmost columns show simulated EFR for the mean audiogram of the NH (left) and HI listeners (middle), respectively. Panels i-j in the rightmost column show the corresponding EFR_{AN} magnitude-level functions obtained by summing up all AN activity across CF. Blue circles show NH simulations and red diamonds represent HI simulations. The thin solid lines represent noise floor estimates. Only OHC dysfunction was assumed to adjust the AN model parameters to account for the HI mean audiogram values.

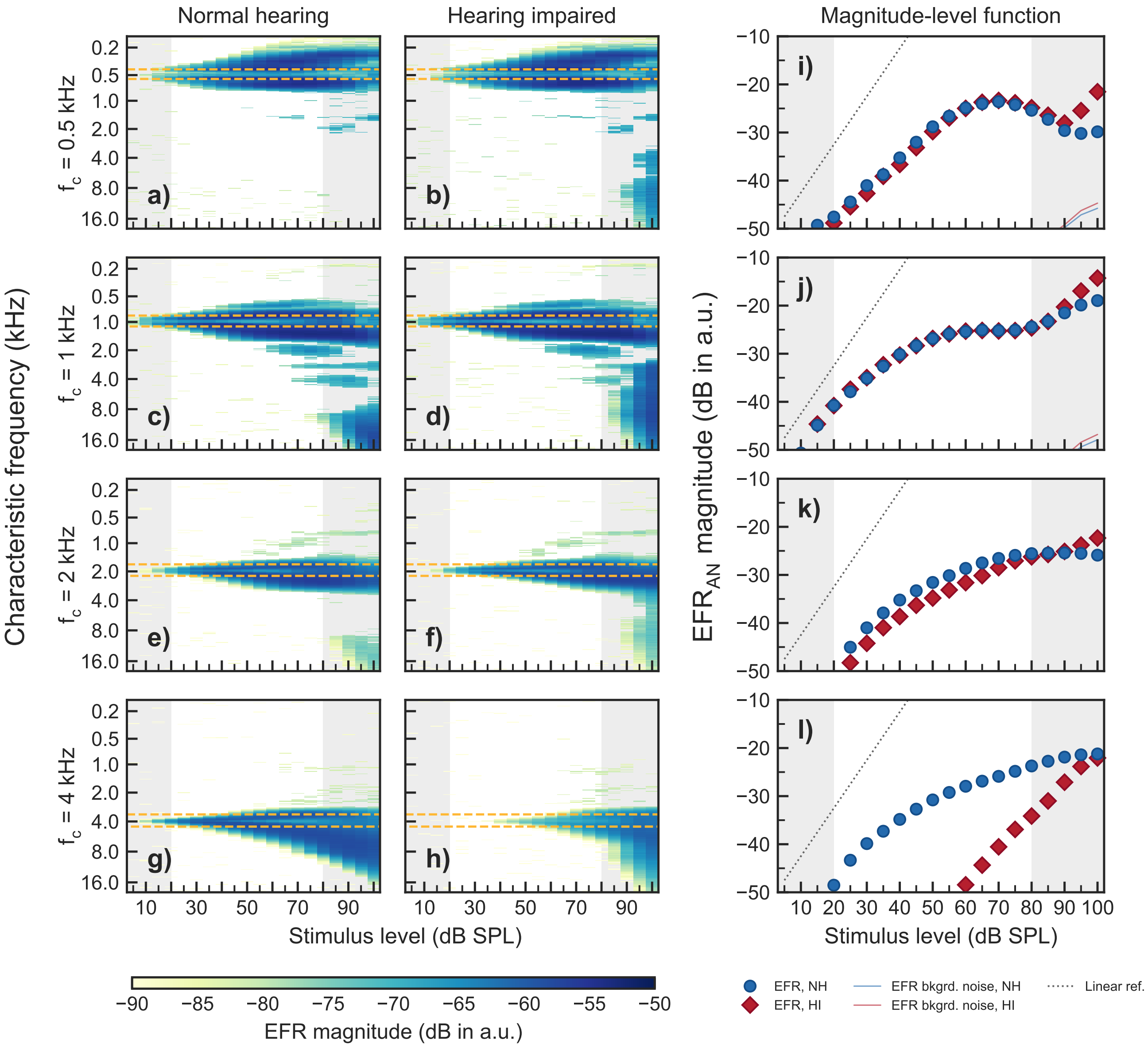

Supplementary Figure 5. Simulated EFR at the level of the AN (EFR_{AN}) obtained with four simultaneously presented SAM tones as the ones used in the experiments for NH and HI assuming only IHC dysfunction. Same representation as in Supplementary Fig. 4 but assuming only IHC dysfunction to adjust the AN model parameters to account for the HI mean audiogram values.

Supplementary Table 1. Fitted parameters to the EFR magnitude-level functions for all NH listeners at all frequencies. The values in the brackets indicate the lower and upper 95 % CI.

	Frequency			
Listener	500 Hz	1000 Hz	2000 Hz	4000 Hz
NH01	s_1 = 0.49 (0.40, 0.57)<br>s_2 = -0.02 (-0.47, 0.42)<br>b_x = 65.0 (54.1, 75.9)<br>b_y = -18.6 (-23.2, -14.1)	s_1 = 0.52 (0.40, 0.64)<br>s_2 = -0.16 (-0.57, 0.25)<br>b_x = 61.8 (53.4, 70.3)<br>b_y = -16.0 (-19.8, -12.1)	s_1 = 0.37 (0.28, 0.46)<br>s_2 = -0.01 (-0.13, 0.11)<br>b_x = 53.8 (46.6, 60.8)<br>b_y = -22.4 (-24.2, -20.6)	s = 0.33 (0.29, 0.37)<br>b = -43.5 (-45.7, -41.3)
NH02	s_1 = 0.71 (-0.04, 1.46)<br>s_2 = -0.01 (-021, 0.20)<br>b_x = 41.7 (31.6, 51.9)<br>b_y = -28.4 (-32.6, -24.3)	s_1 = 0.42 (0.17, 0.67)<br>s_2 = 0.04 (-0.16, 0.24)<br>b_x = 50.0 (33.9, 66.1)<br>b_y = -24.8 (-28.8, -20.8)	s_1 = 0.38 (0.22, 0.54)<br>s_2 = -0.11 (-0.43, 0.22)<br>b_x = 57.0 (44.6, 69.3)<br>b_y = -22.2 (-26.1, -18.4)	s = 0.17 (0.11, 0.23)<br>b = -39.2 (-42.4, -36.0)
NH03	s = 0.24 (0.08, 0.40)<br>b = -41.3 (-51.0, -31.7)	s_1 = 0.77 (0.12, 1.42)<br>s_2 = 0.18 (0.11, 0.25)<br>b_x = 43.0 (35.8, 50.1)<br>b_y = -28.9 (-31.3, -26.4)	s = 0.32 (0.24, 0.40)<br>b = -47.6 (-52.2, -42.9)	s = 0.31 (-0.10, 0.72)<br>b = -47.7 (-71.3, -24.1)
NH04	s = 0.19 (0.12, 0.26)<br>b = -41.1 (-45.3, -36.8)	s = 0.16 (0.07, 0.25)<br>b = -39.9 (-45.7, -34.2)	s = 0.10 (0.00, 0.20)<br>b = -39.3 (-45.7, -32.8)	s = 0.18 (-0.30, 0.67)<br>b = -44.6 (-78.1, -11.0)
NH05	s_1 = 0.45 (0.11, 0.78)<br>s_2 = 0.17 (0.05, 0.28)<br>b_x = 55.0 (40.7, 69.3)<br>b_y = -27.0 (-30.5, -23.6)	s_1 = 0.37 (0.19, 0.56)<br>s_2 = 0.01 (-0.23, 0.25)<br>b_x = 56.0 (42.7, 69.3)<br>b_y = -23.2 (-27.1, -19.3)	s_1 = 0.29 (0.15, 0.42)<br>s_2 = -0.28 (-0.60, 0.03)<br>b_x = 53.8 (46.6, 60.8)<br>b_y = -21.6 (-23.9, -19.3)	s = 0.24 (0.15, 0.32)<br>b = -39.5 (-44.3, -34.7)
NH06	s = 0.20 (0.03, 0.37)<br>b = -42.9 (-54.0, -31.9)	s_1 = 0.38 (0.30, 0.47)<br>s_2 = -0.02 (-0.13, 0.09)<br>b_x = 51.6 (45.2, 58.0)<br>b_y = -25.4 (-27.2, -23.5)	s_1 = 0.31 (0.13, 0.49)<br>s_2 = -0.48 (-1.31, 0.35)<br>b_x = 64.9 (56.1, 73.7)<br>b_y = -29.3 (-32.7, -25.8)	s = 0.16 (0.09, 0.24)<br>b = -42.1 (-46.8, -37.4)
NH07	s = 0.13 (-0.39, 0.66)<br>b = -36.3 (-68.3, -4.3)	s_1 = 0.21 (0.16, 0.25)<br>s_2 = -0.24 (-0.47, -0.02)<br>b_x = 69.4 (65.0, 73.7)<br>b_y = -23.2 (-24.2, -22.2)	s_1 = 0.20 (0.09, 0.32)<br>s_2 = -0.11 (-0.76, 0.56)<br>b_x = 70.0 (49.7, 90.3)<br>b_y = -23.8 (-26.9, -20.6)	s = 0.18 (0.13, 0.24)<br>b = -39.6 (-42.5, -36.6)
NH08	s = 0.24 (0.01, 0.46)<br>b = -41.3 (-55.3, -27.2)	s = 0.24 (0.19, 0.28)<br>b = -41.1 (-43.6, -38.6)	s = 0.22 (0.14, 0.30)<br>b = -39.2 (-43.7, -34.7)	s = 0.21 (0.15, 0.28)<br>b = -41.8 (-45.6, -38.0)
NH09	s = 0.19 (0.06, 0.32)<br>b = -39.3 (-47.7, -30.9)	s_1 = 0.86 (0.17, 1.55)<br>s_2 = -0.13 (-0.44, 0.18)<br>b_x = 57.7 (50.2, 65.2)<br>b_y = -22.1 (-26.0, -18.2)	s_1 = 0.21 (0.14, 0.28)<br>s_2 = -0.17 (-0.39, 0.03)<br>b_x = 62.5 (55.0, 70.0)<br>b_y = -25.4 (-26.9, -23.8)	s = 0.17 (0.06, 0.28)<br>b = -36.7 (-43.5, -29.9)
NH10	s_1 = 0.61 (0.22, 1.01)<br>s_2 = 0.18 (0.02, 0.34)<br>b_x = 46.3 (34.2, 58.5)<br>b_y = -26.2 (-31.3, -21.1)	s_1 = 0.31 (0.26, 0.37)<br>s_2 = -0.08 (-0.25, 0.09)<br>b_x = 63.7 (58.0, 69.4)<br>b_y = -18.1 (-29.6, -16.7)	s = 0.20 (0.10, 0.29)<br>b = -38.1 (-43.7, -32.5)	s = 0.26 (0.16, 0.36)<br>b = -41.7 (-47.5, -35.9)
NH11	s = 0.19 (0.04, 0.34)<br>b = -39.7 (-49.2, -30.2)	s = 0.15 (0.04, 0.26)<br>b = -35.7 (-42.9, -28.6)	s = 0.04 (-0.07, 0.16)<br>b = -30.5 (-37.8, -23.2)	s = 0.19 (0.05, 0.34)<br>b = -40.0 (-49.2, -30.9)
NH12	s = 0.26 (0.23, 0.28)<br>b = -46.4 (-48.0, -44.8)	s = 0.25 (0.14, 0.36)<br>b = -43.8 (-49.9, -37.7)	s = 0.25 (0.14, 0.37)<br>b = -43.6 (-50.7, -36.5)	s = 0.31 (0.25, 0.37)<br>b = -47.5 (-51.1, -43.8)
NH13	s_1 = 0.28 (0.20, 0.35)<br>s_2 = 0.00 (-0.25, 0.24)<br>b_x = 63.6 (51.9, 75.4)<br>b_y = -26.4 (-29.3, -23.5)	s = 0.24 (0.11, 0.38)<br>b = -39.4 (-48.2, -30.7)	s_1 = 0.58 (0.38, 0.78)<br>s_2 = 0.00 (-0.20, 0.20)<br>b_x = 60.0 (54.4, 65.6)<br>b_y = -24.6 (-27.3, -21.9)	s = 0.31 (0.19, 0.43)<br>b = -45.8 (-53.8, -37.9)

Supplementary Table 2. Fitted parameters to the EFR magnitude-level functions for all HI listeners at all frequencies, including lower and upper 95 % CI in brackets.

Listener	Frequency 500 Hz	Frequency 1000 Hz	Frequency 2000 Hz	Frequency 4000 Hz
HI01	s_1 = 0.40 (0.32, 0.47)<br>s_2 = -0.04 (-0.17, 0.09)<br>b_x = 56.5 (50.9, 62.1)<br>b_y = -17.9 (-19.7, -16.1)	s_1 = 0.27 (0.16, 0.37)<br>s_2 = -0.18 (-0.32, -0.05)<br>b_x = 52.7 (45.7, 59.6)<br>b_y = -18.2 (-19.9, -16.6)	s_1 = 0.44 (0.34, 0.54)<br>s_2 = 0.03 (-0.05, 0.11)<br>b_x = 50.0 (44.2, 55.8)<br>b_y = -21.3 (-22.8, -19.8)	s = 0.57 (0.18, 0.95)<br>b = -64.8 (-93.0, -36.5)
HI02	s_1 = 0.64 (0.35, 0.93)<br>s_2 = -0.52 (-1.83, 0.79)<br>b_x = 70.0 (60.6, 79.4)<br>b_y = -24.8 (-30.7, -18.9)	s = 0.35 (0.00, 0.69)<br>b = -52.3 (-76.3, -28.3)	s = 0.30 (0.04, 0.55)<br>b = -51.4 (-68.7, -34.1)	s = 0.25 (-1.96, 2.46)<br>b = -48.8 (-211.5, 114.0)
HI03	s_1 = 0.42 (0.31, 0.52)<br>s_2 = -0.72 (-0.96, -0.48)<br>b_x = 67.3 (65.0, 69.6)<br>b_y = -22.2 (-23.4, -20.9)	s_1 = 0.52 (0.04, 0.99)<br>s_2 = -0.02 (-0.23, 0.20)<br>b_x = 59.2 (48.9, 69.5)<br>b_y = -22.7 (-25.3, -20.0)	s = 0.15 (0.04, 0.26)<br>b = -35.2 (-42.1, -28.2)	s = 0.27 (0.01, 0.52)<br>b = -48.2 (-65.7, -30.6)
HI04	s_1 = 0.56 (0.11, 1.01)<br>s_2 = -0.14 (-0.38, 0.10)<br>b_x = 54.7 (44.5, 64.8)<br>b_y = -28.2 (-31.4, -25.0)	s_1 = 0.31 (0.26, 0.37)<br>s_2 = -0.07 (-0.14, 0.01)<br>b_x = 57.8 (54.0, 61.5)<br>b_y = -23.7 (-24.5, -22.8)	s_1 = 0.41 (0.36, 0.47)<br>s_2 = 0.04 (-0.03, 0.12)<br>b_x = 55.6 (51.7, 59.6)<br>b_y = -25.7 (-27.0, -24.4)	s = 0.41 (0.26, 0.57)<br>b = -58.1 (-68.3, -47.9)
HI05	s = 0.13 (-0.83, 1.09)<br>b = -34.6 (-99.8, 30.6)	s = 0.33 (-0.11, 0.77)<br>b = -39.3 (-57.4, -21.3)		s = 0.50 (-3.32, 4.33)<br>b = -61.9 (-349.2, 225.4)
HI06	s = 0.16 (-0.30, 0.61)<br>b = -34.4 (-64.6, -4.2)	s = 0.53 (0.38, 0.68)<br>b = -53.7 (-61.0, -46.5)	s = 0.19 (-0.02, 0.40)<br>b = -35.3 (-46.7, -23.9)	
HI07	s = 0.26 (-0.31, 0.82)<br>b = -45.8 (-85.0, -6.6)	s = 0.17 (0.06, 0.28)<br>b = -38.7 (-45.1, -32.2)	s = 0.10 (-0.05, 0.24)<br>b = -34.9 (-43.4, -26.4)	

Supplementary Table 3. Fitted parameters to the DPOAE magnitude-level functions for all NH listeners at all frequencies, including lower and upper 95 % CI in brackets.

Listener	Frequency			
	500 Hz	1000 Hz	2000 Hz	4000 Hz
NH01	s = 0.27 (0.11, 0.44)<br>b = -18.0 (-26.1, -10.0)	s = 0.69 (0.38, 1.00)<br>b = -39.8 (-54.9, -24.6)	s = 0.62 (0.23, 1.02)<br>b = -47.7 (-67.0, -28.4)	s = 0.95 (0.80, 1.10)<br>b = -57.8 (-65.0, -50.7)
NH02	s = 0.49 (-0.09, 1.07)<br>b = -19.2 (-47.7, 9.2)	s = 0.72 (0.40, 1.05)<br>b = -43.7 (-59.7, -27.8)	s = 0.85 (0.40, 1.31)<br>b = -52.4 (-74.6, -30.1)	s = 1.05 (0.93, 1.16)<br>b = -56.8 (-62.3, -51.2)
NH04	s = 0.44 (0.18, 0.70)<br>b = -31.2 (-45.1, -17.2)	s = 0.54 (0.43, 0.66)<br>b = -45.5 (-51.1, -39.9)	s = 0.37 (-0.09, 0.82)<br>b = -44.3 (-66.3, -22.3)	s = 0.79 (0.51, 1.07)<br>b = -54.0 (-67.5, -40.4)
NH05	s = 0.57 (0.49, 0.66)<br>b = -35.8 (-40.1, -31.5)	s = 0.75 (0.57, 0.93)<br>b = -44.4 (-53.4, -35.5)	s = 0.81 (0.60, 1.03)<br>b = -51.6 (-62.0, -41.1)	s = 0.92 (0.54, 1.30)<br>b = -61.5 (-80.0, -43.0)
NH06	s = 0.52 (-0.06, 1.10)<br>b = -32.5 (-62.0, -2.97)	s = 0.86 (0.73, 0.99)<br>b = -46.6 (-52.8, -40.4)	s = 0.60 (0.28, 0.92)<br>b = -48.0 (-63.7, -32.3)	s = 0.49 (0.23, 0.75)<br>b = -47.6 (-60.3, -35.0)
NH07	s = 0.33 (0.08, 0.58)<br>b = -19.5 (-31.7, -7.3)	s = 0.98 (0.61, 1.36)<br>b = -51.6 (-69.7, -33.4)	s = 0.42 (0.34, 0.50)<br>b = -25.4 (-29.4, -21.4)	s = 0.83 (0.67, 0.98)<br>b = -49.4 (-57.0, -41.8)
NH08	s = 0.43 (-0.09, 0.94)<br>b = -25.3 (-51.2, 0.57)	s = 0.22 (-0.19, 0.63)<br>b = -24.5 (-44.7, -4.3)	s = 0.40 (0.08, 0.72)<br>b = -44.2 (-59.9, -28.5)	s = 0.12 (-0.41, 0.64)<br>b = -31.8 (-57.5, -6.0)
NH09	s = 0.52 (0.34, 0.70)<br>b = -32.8 (-42.1, -23.6)	s = 0.72 (0.57, 0.86)<br>b = -42.0 (-49.3, -34.8)	s = 0.40 (0.17, 0.63)<br>b = -38.6 (-49.8, -27.4)	s = 0.79 (0.56, 1.01)<br>b = -56.7 (-67.6, -45.7)
NH10	s = 0.53 (0.24, 0.82)<br>b = -33.3 (-48.6, -18.1)	s = 0.93 (0.51, 1.34)<br>b = -54.1 (-74.5, -33.7)	s = 0.39 (0.10, 0.68)<br>b = -40.6 (-54.8, -26.5)	s = 0.46 (0.08, 0.85)<br>b = -43.7 (-62.3, -25.1)
NH11	s = 0.47 (0.19, 0.76)<br>b = -31.3 (-45.6, -17.0)	s = 0.75 (0.62, 0.88)<br>b = -52.1 (-58.6, -45.7)	s = 0.47 (0.22, 0.72)<br>b = -43.4 (-55.5, -31.2)	s = 0.44 (-0.05, 0.94)<br>b = -42.6 (-67.4, -17.9)
NH12	s = 0.67 (0.48, 0.87)<br>b = -42.3 (-53.3, -31.4)	s = 0.62 (0.21, 1.03)<br>b = -45.9 (-66.0, -25.9)	s = 0.53 (0.31, 0.75)<br>b = -43.9 (-54.6, -33.2)	s = 0.29 (0.04, 0.53)<br>b = -41.0 (-52.8, -29.3)
NH13	s = 0.63 (0.39, 0.86)<br>b = -28.1 (-39.6, -16.6)	s = 0.97 (0.75, 1.19)<br>b = -52.9 (-63.6, -42.2)	s = 0.40 (0.11, 0.68)<br>b = -39.0 (-52.9, -25.1)	s = 0.65 (0.26, 1.04)<br>b = -49.6 (-68.6, -30.7)
